# The clinical implication of minimally invasive versus open pancreatoduodenectomy for non-pancreatic periampullary cancer: a systematic review and individual patient data meta-analysis

**DOI:** 10.1007/s00423-023-03047-4

**Published:** 2023-08-15

**Authors:** Bas A. Uijterwijk, Meidai Kasai, Daniel H. L. Lemmers, Palanivelu Chinnusamy, Jony van Hilst, Benedetto Ielpo, Kongyuan Wei, Ki Byung Song, Song C. Kim, Sjors Klompmaker, Jin-Young Jang, Kelly M. Herremans, Lapo Bencini, Andrea Coratti, Michele Mazzola, Krishna V. Menon, Brian K. P. Goh, Renyi Qin, Marc G. Besselink, Mohammed Abu Hilal, for the International Study Group on non-pancreatic periAmpullary CAncer (ISGACA), Abdul Hakeem, Fernando Burdio, Palanisamy Senthilnathan, Patricia Sánchez, Hyeong Seok Kim, Steven J. Hughes, Alessandro Giani

**Affiliations:** 1 Department of Surgery, Fondazione Poliambulanza Istituto Ospedaliero, Brescia, Italy; 2 Department of Surgery, Amsterdam UMC, Location University of Amsterdam, Amsterdam, the Netherlands; 3 Department of Surgery, Meiwa Hospital, Hyogo, Japan; 4 Department of Surgical Gastroenterology and Hepatopancreatobiliary Surgery, GEM Hospital and Research Center, Ramanathapuram, Coimbatore, Tamil Nadu India; 5 Department of Surgery, OLVG, Amsterdam, the Netherlands; 6 Hepatobiliary and Pancreatic Surgery Unit, Hospital del Mar. Universitat Pompeu Fabra, Barcelona, Spain; 7 Department of General, Visceral and Transplantation Surgery, University of Heidelberg, Heidelberg, Germany; 8 Division of Hepatobiliary and Pancreatic Surgery, Department of Surgery, University of Ulsan College of Medicine and Asan Medical Center, Seoul, Korea; 9 Department of Surgery and Cancer Research Institute, Seoul National University College of Medicine, Seoul, South Korea; 10 Division of Surgical Oncology, General Surgery, University of Florida, Gainesville, USA; 11 Department of Surgery, Careggi University Hospital, Florence, Italy; 12 Department of Surgery, Misericordia Hospital of Grosseto, Grosseto, Italy; 13 Division of Oncologic and Mini-Invasive General Surgery, ASST Grande Ospedale Metropolitano Niguarda, Milan, Italy; 14 Department of Liver Transplant and HPB Unit, King’s College Hospital, London, UK; 15 Department of Hepatopancreatobiliary and Transplant Surgery, Singapore General Hospital, Singapore, Singapore; 16 Department of Biliary-Pancreatic Surgery, Affiliated Tongji Hospital, Tongji Medical College, Huazhong University of Science and Technology, Wuhan, China

**Keywords:** Minimally invasive surgery, Minimally invasive pancreatoduodenectomy, Ampulla of Vater carcinoma, Duodenal adenocarcinoma, Distal cholangiocarcinoma, Individual patient data meta-analysis

## Abstract

**Background:**

Most studies on minimally invasive pancreatoduodenectomy (MIPD) combine patients with pancreatic and periampullary cancers even though there is substantial heterogeneity between these tumors. Therefore, this study aimed to evaluate the role of MIPD compared to open pancreatoduodenectomy (OPD) in patients with non-pancreatic periampullary cancer (NPPC).

**Methods:**

A systematic review of Pubmed, Embase, and Cochrane databases was performed by two independent reviewers to identify studies comparing MIPD and OPD for NPPC (ampullary, distal cholangio, and duodenal adenocarcinoma) (01/2015–12/2021). Individual patient data were required from all identified studies. Primary outcomes were (90-day) mortality, and major morbidity (Clavien-Dindo 3a-5). Secondary outcomes were postoperative pancreatic fistula (POPF), delayed gastric emptying (DGE), postpancreatectomy hemorrhage (PPH), blood-loss, length of hospital stay (LOS), and overall survival (OS).

**Results:**

Overall, 16 studies with 1949 patients were included, combining 928 patients with ampullary, 526 with distal cholangio, and 461 with duodenal cancer. In total, 902 (46.3%) patients underwent MIPD, and 1047 (53.7%) patients underwent OPD. The rates of 90-day mortality, major morbidity, POPF, DGE, PPH, blood-loss, and length of hospital stay did not differ between MIPD and OPD. Operation time was 67 min longer in the MIPD group (*P* = 0.009). A decrease in DFS for ampullary (HR 2.27, *P* = 0.019) and distal cholangio (HR 1.84, *P* = 0.025) cancer, as well as a decrease in OS for distal cholangio (HR 1.71, *P* = 0.045) and duodenal cancer (HR 4.59, *P* < 0.001) was found in the MIPD group.

**Conclusions:**

This individual patient data meta-analysis of MIPD versus OPD in patients with NPPC suggests that MIPD is not inferior in terms of short-term morbidity and mortality. Several major limitations in long-term data highlight a research gap that should be studied in prospective maintained international registries or randomized studies for ampullary, distal cholangio, and duodenum cancer separately.

**Protocol registration:**

PROSPERO (CRD42021277495) on the 25th of October 2021.

**Supplementary Information:**

The online version contains supplementary material available at 10.1007/s00423-023-03047-4.

## Introduction

Periampullary cancer is a widely used term to define a heterogeneous group of neoplasms in the pancreatic head, the ampulla of Vater, distal bile duct, and duodenum [1, 2]. Pancreatic ductal adenocarcinoma (PDAC) is the most frequently diagnosed periampullary cancer and also has the worst prognosis with 5-year overall survival of 5–22% after surgical resection [3–6]. The remaining periampullary tumors are commonly classified into a single category of non-pancreatic periampullary cancer (NPPC) [7]. Despite anatomic similarities, there are fundamental and biological differences between the NPPCs [8–12]. The distinct origin is associated with a variety of reported 5-year survival, ranging between 30 to 70% for ampullary [13–16] cancer, 18 to 40% for distal cholangiocarcinoma [17–20], and 46 to 71% for duodenal cancer [21–24].

After diagnosis with resectable NPPC (ampullary, distal cholangio, or duodenal cancer), the only curative treatment is a pancreatoduodenectomy. In the last decade, minimally invasive pancreatoduodenectomy (MIPD) has been widely implemented, both for PDAC and NPPC [25], and a shift is taking place from traditional open pancreatoduodenectomy (OPD) towards MIPD [26–28]. Some studies have demonstrated potential peri- and postoperative benefits of the MIPD when compared to the traditional OPD, including shorter hospital stay, decreased intraoperative blood-loss, and lower rates of wound infections [29–33]. However, despite the fundamental heterogeneity of the various NPPCs, the majority of the studies on MIPD assessed periampullary lesions as one single entity (Fig. A1, Appendix). This can lead to important disharmony in the compared groups and hence, inaccurate treatment strategies in clinical practice.

The primary aim of the present study was to compare the mortality and major morbidity after MIPD and OPD in patients with NPPC and its subgroups using individual patient-level data from published studies, identified by a systematic literature review. By collecting all relevant evidence on the topic, this study can assist in determining the best surgical strategy and potentially guide clinical decision making in the treatment of ampullary, distal cholangio, and duodenum cancer.

## Methods

### Design

This study was designed as a systematic review and individual patient data meta-analysis (IPDMA). This study was conducted in accordance with the Preferred Reporting Items for Systematic Reviews and Meta-Analyses statement (PRISMA-IPD [34]) and Assessing the Methodological Quality of Systematic Reviews (AMSTAR [35]) guidelines. The protocol was developed before the start of the reviewing process registered in the online openly available PROSPERO registry.

### Study identification, search strategy, and selection criteria

All comparative studies (retrospective cohort, prospective cohort, and randomized controlled trial) comparing MIPD with OPD for periampullary tumors were identified using Pubmed, Embase (via Ovid), and Cochrane databases. The keywords “minimally invasive,” “laparoscopic,” “robotic,” “open,” “pancreatoduodenectomy,” and synonyms were used to identify all relevant studies from January 2015 until December 2021 (full search available in the Appendix). Following exclusion of duplicates, two authors (BAU and DHL) independently reviewed the titles, abstracts, and full texts of studies identified by the literature search. Studies reporting a comparison of minimally invasive and open pylorus-resecting or -preserving pancreatoduodenectomy in human subjects for periampullary cancers including ampullary, distal cholangio, duodenal cancer, and written in English were included. Excluded were (1) studies with a non-comparative cohort design (e.g., review articles, case reports, technical procedure reports, pilot trials), (2) studies evaluating different types of pancreatic surgery (e.g., distal/total pancreatectomy), or (3) studies selectively focusing on PDAC or benign indications (e.g., chronic pancreatitis or benign tumors). The search was extended with a manual evaluation of relevant references used in the included articles. Conflicts or concerns were resolved by discussion between the two reviewers and a third reviewer (MAH). After reaching consensus of the included studies, all corresponding authors or principal investigators were contacted with the study protocol describing the objectives and procedures of this IPDMA, and the database used for the selected study was requested.

### Inclusion criteria

From the received databases, only the cases with ampullary, distal cholangio, and duodenal cancers (NPPCs) operated with pylorus-preserving pancreatoduodenectomy (Whipple’s procedure) or pylorus-resecting pancreatoduodenectomy were included for analyses. Patients with PDAC, benign neoplasms, hybrid procedures, cases with missing primary outcomes, or operated with other surgical techniques (e.g., total pancreatectomy, duodenum sparing pancreatectomy, ampullectomy) were excluded from final analyses.

### Outcomes

The primary outcomes were 90-day mortality and postoperative major morbidity, defined as Clavien-Dindo 3a-5 [36]. Secondary outcomes were operation time (minutes), perioperative estimated blood-loss (ml), postoperative incidence of postoperative pancreatic fistula (POPF), delayed gastric emptying (DGE), postpancreatectomy hemorrhage (PPH), wound infections, and the length of hospital stay (days). Oncological outcomes included R1 resections, disease-free survival (DFS), and overall survival (OS). The 3-year survival and recurrence were reported in the percentage of the patients “at risk” 36 months after surgery.

### Subgroup analyses

Using subgroup analyses, MIPD and OPD were compared for ampullary, distal cholangio, and duodenal cancer separately. Other subgroup analyses we considered to be relevant were (1) young versus elderly patients, since perioperative blood-loss and complication rates are known to be higher in elderly patients [37], (2) pylorus-resecting versus pylorus-preserving pancreatoduodenectomy, since pylorus-preserving pancreatoduodenectomy is known to be associated with higher rates of DGE [38], and (3) outcomes between Western (European-USA) centers and Eastern (Asian-Pacific) centers because in Asia–Pacific centers, it is common practice to discharge a patient to their pre-surgical living situation. In European centers, it is more common to discharge a patient to a temporary stay in a rehabilitation center, and in American centers, there is a well-organized home-nursing system in place which could offer postoperative care at home. Since patients must be in a better state of recovery before returning to their previous living situation, the length of hospital stays in different continents could correlate with a different stage of rehabilitation. Additionally, patient population in Asian-Pacific centers are known to have a lower BMI that could be a predictive factor for postoperative complications or survival [39].

### Definitions

NPPC is defined as adenocarcinoma arising from the ampulla of Vater, distal bile duct, and periampullary duodenum (second segment), extracted from postoperative pathology report. ASA classification was defined following American Society of Anesthesiologists (ASA) classification [40]. TNM staging was according to the 7th and 8th editions of the American Joint Committee on Cancer (AJCC) [41, 42]. Since N2 stage is only introduced in the 8th edition, the N2 tumors in the 8th edition would have been N1 in the 7th edition. Therefore, N-stage is classified in N0 and N1-2. POPF, DGE, and PPH were defined as ISGPS grade B and C [43–45]. An R1 resection margin is defined as < 1 mm according to the definition of the Royal College of Pathologists [46]. All converted cases were included in the minimally invasive group following the intention to treat principle.

### Statistical analyses

Normally distributed variables are reported as means with standard deviation (SD). Non-normally distributed variables are reported as medians with interquartile ranges (IQR). Categorical variables are presented as frequencies and proportions. Categorical data were compared by means of the chi square test, whereas numerical data were compared by the Student *t*-test for normally distributed data and non-normally distributed data by its non-parametric equivalent the Mann–Whitney *U* test. Standardized mean differences (SMD) were used to assess balance. An SMD below 0.2 is deemed as optimal balance [47, 48]. The meta-analysis was performed using R (version 3.6.1; the R Foundation for Statistical Computing, Vienna, Austria). Statistical significance was set at *P* < 0.05, with 95% confidence intervals (CI) shown for all results. A “2-stage” approach was adopted for the IPDMA, as recommended; the IPD within studies generated summary measures, which were combined using standard meta-analytical methods. The fixed-effects model was adopted if heterogeneity was not statistically significant. The random-effects model was used when statistical heterogeneity was identified. The mean difference (MD) in continuous variables was compared using the inverse variance method, and categorical dichotomous variables were assessed using risk differences (RDs) by the inverse variance method. OS was assessed using the hazard ratio (HR) which was calculated using the Cox proportional hazard model. Heterogeneity was assessed using Cochran’s Q test, the observed values of *I*^2^ were used to represent the severity of heterogeneity and were interpreted using thresholds that were previously recommended (0–40%: likely minimal; 30–60%: likely moderate; 50–90%: likely substantial; and 75–100%: likely considerable), along with the strength of evidence [49]. Funnel plots were used to explore the presence of publication bias visually, and their symmetry was evaluated by the Egger’s test [50].

## Results

### Systematic review

**Fig. 1:**
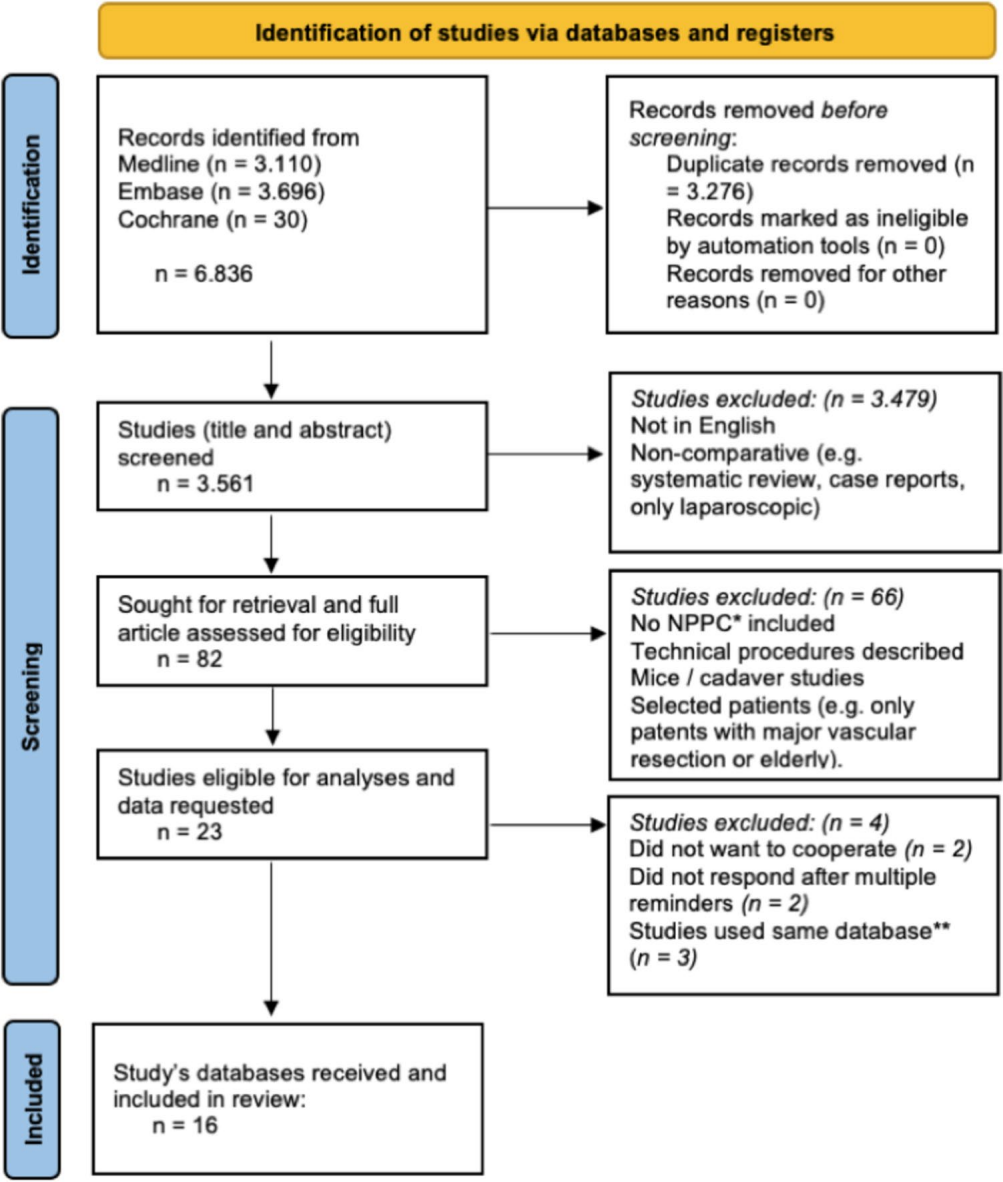
Flowchart systematic web search; *NPPC, non-pancreatic periampullary cancer; **when multiple studies used the same database, the complete database of the most recent study was requested

Of the 3530 screened studies, 23 compared MIPD versus OPD with included NPPC and matched the selection criteria (Fig. 1). Subsequently, the corresponding or senior authors were invited to share the database of their study. When multiple studies were based on the same database, the most complete database of the most recent study was requested [51–53]. Requested databases of four studies were not retrieved [54–57].

### Study and patient characteristics

In total, 16 studies, of which four randomized controlled trials and 12 retrospective cohort studies, were included [29, 33, 51, 52, 58–69]. Included number of patients with NPPC varied between 14 [60] and 436 [29] per study (Table 1). One study [51] focused on ampullary cancer in specific, while the remaining 15 studies included all NPPCs. Most studies were conducted in South Korea, China, and the Netherlands (World map, Fig. A2, Appendix). No conflicts of interests were identified in the included studies.

**Table 1:** Characteristics of the included studies

Study	Country	Study design	No. of patients			Indication		
			Total	MIPD	OPD	MIPD	OPD	*P-value*
Hakeem et al. 2014 [58]	UK	RCS	24	12	12	AAC, 9 (38%)	AAC, 9 (38%)	*1.000*
						DCC, 3 (13%)	DCC, 3 (13%)	
						DAC, 0 (0%)	DAC, 0 (0%)	
Low et al. 2020 [52]	Singapore	RCS	14	5	9	AAC, 4 (33%)	AAC, 4 (33%)	*0.598*
						DCC, 2 (17%)	DCC, 1 (8%)	
						DAC, 1 (8%)	DAC, 0 (0%)	
van Hilst et al. 2019 [59]	Netherlands	RCT	38	20	18	AAC, 12 (32%)	AAC, 6 (16%)	*0.254*
						DCC, 5 (13%)	DCC, 8 (21%)	
						DAC, 3 (8%)	DAC, 4 (11%)	
Poves et al. 2017 [60]	Spain	RCT	14	11	3	AAC, 5 (36%)	AAC, 1 (7%)	*0.707*
						DCC, 6 (43%)	DCC, 2 (14%)	
						DAC, 0 (0%)	DAC, 0 (0%)	
Mazzola et al. 2021 [61]	Italy	RCS	27	16	11	AAC, 11 (41%)	AAC, 6 (22%)	*0.424*
						DCC, 5 (19%)	DCC, 4 (15%)	
						DAC, 0 (0%)	DAC, 1 (4%)	
Palanivelu et al. 2019 [62]	India	RCT	53	29	24	AAC, 15 (28%)	AAC, 11 (21%)	*0.582*
						DCC, 4 (8%)	DCC, 6 (11%)	
						DAC, 10 (19%)	DAC, 7 (13%)	
Shin et al. 2019 [63]	South Korea	RCS	80	40	39	AAC, 19 (24%)	AAC, 17 (22%)	*0.127*
						DCC, 19 (24%)	DCC, 20 (25%)	
						DAC, 4 (5%)	DAC, 0 (0%)	
Yoo et al. 2020 [77]	South Korea	RCS	359	76	282	AAC, 76 (21%)	AAC, 282 (79%)	*0.604*
						DCC, 0	DCC, 0	
						DAC, 0	DAC, 0	
Klompmaker et al. 2018 [64]	Netherlands	RCS	308	266	42	AAC, 131 (43%)	AAC, 25 (8%)	*P* = *0.463*
						DCC, 89 (29%)	DCC, 11 (4%)	
						DAC, 46 (15%)	DAC, 6 (2%)	
Kim et al. 2021 [33]	South Korea	RCS	178	45	133	AAC, 22 (12%)	AAC, 66 (37%)	*0.939*
						DCC, 20 (11%)	DCC, 60 (34%)	
						DAC, 3 (2%)	DAC, 7 (4%)	
Bencini et al. 2020 [65]	Italy	RCS	45	23	22	AAC, 17 (38%)	AAC, 6 (13%)	***0.007***
						DCC, 5 (11%)	DCC, 14 (31%)	
						DAC, 1 (2%)	DAC, 2 (4%)	
Deichmann et al. 2016 [66]	Germany	RCS	31	14	17	AAC, 7 (23%)	AAC, 9 (29%)	*0.950*
						DCC, 4 (13%)	DCC, 4 (13%)	
						DAC, 3 (10%)	DAC, 4 (13%)	
Choi et al. 2020 [67]	South Korea	RCS	118	69	49	AAC, 31 (26%)	AAC, 9 (8%)	***0.010***
						DCC, 34 (29%)	DCC, (37 (31%)	
						DAC, 4 (3%)	DAC, 3 (3%)	
Delitto et al. 2014 [68]	USA	RCS	52	24	28	AAC, 13 (25%)	AAC, 17 (33%)	*0.483*
						DCC, 10 (19%)	DCC, 8 (15%)	
						DAC, 1 (2%)	DAC, 3 (6%)	
Wang et al. 2021 [69]	China	RCT	173	100	73	AAC, 16 (9%)	AAC, 13 (8%)	*0.907*
						DCC, 40 (23%)	DCC, 27 (16%)	
						DAC, 44 (25%)	DAC, 33 (19%)	
Dang et al. 2021 [29]	China	RCS	436	148	288	AAC, 24 (6%)	AAC, 51 (12%)	*0.324*
						DCC, 34 (8%)	DCC, 49 (11%)	
						DAC, 90 (21%)	DAC, 188 (43%)	

Bold values indicate a significance level <0.05

Abbreviations: *MIPD* minimally invasive pancreatoduodenectomy, *OPD* open pancreatoduodenectomy, *RCS* retrospective cohort study, *RCT* randomized controlled trial, *AAC* ampullary adenocarcinoma, *DCC* distal cholangiocarcinoma, *DAC* duodenal adenocarcinoma

The meta-analyses included a total of 1949 patients with resected NPPC, consisting of 902 MIPD and 1047 OPD. Within the MIPD cohort, 146 patients after Robotic PD were included. Patient demographics, surgical details, and tumor characteristics are demonstrated in Table 2, and elaborate baseline characteristics per NPPC subgroup can be found in the Appendix (Table A1). Age and BMI were higher in the MIPD group (65 versus 63 years and 23.2 versus 22.9kg/m^2^, Table 2) and there were less patients with ampullary cancer in the MIPD group (46 versus 51%; *P* = 0.016, Table 2) and more patients with distal cholangiocarcinoma in the MIPD group (31 versus 24%; *P* < 0.001; Table 2). In the MIPD group, more patients with distal cholangio and duodenal cancer had a higher T-stage (*P* = 0.009 and *P* = 0.047 respectively), while the tumor size of only distal cholangiocarcinoma was larger in the OPD group (median 20 versus 25mm; *P* = 0.001, Table 2). Other baseline characteristics (sex, ASA, NPPC subgroup, N-stage, adjuvant chemotherapy, and R1 resection) did not differ significantly between the MIPD and OPD group based on *P*-value and standardized mean differences (Table 2) [47].

**Table 2:** Baseline characteristics of all included patients

	Total (1949)	MIPD (902, 46.3%)	OPD (1047, 53.7%)	*P-*value	*SMD*
Age, yrs, median (IQR)	64 (56–71)	65 (57–72)	63 (55–71)	**0.015**	
Male, *n* (%)	1131 (58)	507 (56.2)	624 (59.6)	0.130	0.016
ASA, mean (SD)	2.1 (0.5)	2.1 (0.6)	2.1 (0.5)	0.395	0.037
BMI, median kg/m^2^ (IQR)	23.4 (3.4)	23.2 (21.0–25.6)	22.9 (21.0–25.1)	**0.032**	
NPPC subtype				**0.003**	0.136
*AAC, n (%)*	*928 (49)*	*403 (46)*	*525 (51)*	***0.016***	*0.110*
*DCC, n (%)*	*526 (28)*	*276 (31)*	*250 (24)*	** < *0.001***	*0.151*
*DAC, n (%)*	*461 (24)*	*207 (23)*	*254 (25)*	*0.500*	*0.031*
Tumor size, median (IQR)	15 (20–30)	20 (15–28)	21 (15–30)	**0.038**	
*AAC, median (IQR)*		*20 (13–25)*	*20 (15–26)*	*0.185*	
*DCC, median (IQR)*		*20 (16–28)*	*25 (20–34)*	***0.001***	
*DAC, median (IQR)*		*20.5 (16.8–35)*	*20 (16–30)*	*0.156*	
T-stage tumor, total, *n* (%)	T1-2: 635 (56) T3-4: 509 (45)	T1-2: 259 (50) T3-4: 257 (50)	T1-2: 376 (60) T3-4: 252 (40)	** < 0.001**	**0.231**
*AAC, n* (%)		*T1-2: 189 (60)* *T3-4: 125 (40)*	*T1-2: 288 (64)* *T3-4: 161 (36)*	0.267	0.082
*DCC, n* (%)		*T1-2: 61 (38)* *T3-4: 100 (62)*	*T1-2: 72 (53)* *T3-4: 64 (47)*	**0.009**	**0.307**
*DAC, n* (%)		*T1-2: 3 (12)* *T3-4: 22 (88)*	*T1-2: 9 (36)* *T3-4: 16 (64)*	**0.047**	**0.580**
N-stage 1–2, *n* (%)	N0: 627 (60) N1-2: 418 (40)	N0: 343 (61) N1-2: 219 (39)	N0: 377 (59) N1-2: 262 (41)	0.480	0.041
Adjuvant Chemotherapy, *n* (%)	412 (41)	179 (43)	233 (40)	0.470	0.046
R1 resection margin, *n* (%)	142 (8)	67 (8)	75 (7)	0.659	0.020
Lymph nodes resected, median (IQR)	14 (10–19)	13 (10–19)	14 (11–20)	** < 0.001**	
*AAC,* median (IQR)		*14 (10–19)*	*15 (10–21)*	*0.113*	
*DCC,* median (IQR)		*13 (9–19)*	*15 (11–20)*	***0.037***	
*DAC,* median (IQR)		*13 (10–16)*	*14 (12–17)*	***0.019***	
PA-positive lymph nodes, median (IQR)	0 (0–2)	0 (0–2)	0 (0–2)	0.926	
Lymph node ratio	0.00 (0.00–0.10)	0.00 (0.00–0.11)	0.00 (0.00–0.10)	0.759	

Bold values indicate a significance level <0.05

Abbreviations: *SD* standard deviation, *n* count, *AC* adenocarcinoma, *OPD* open pancreatoduodenectomy, *MIPD* minimally invasive pancreatoduodenectomy, *SMD* standardized mean difference (not applicable for median and IQR), *AAC* ampullary adenocarcinoma, *DCC* distal cholangiocarcinoma, *DAC* duodenal adenocarcinoma, T-stage tumor: differentiated in T1 and T2 versus T3 and T4 groups (elaborate T-stage distribution in the Appendix table A1), following AJCC 7th and 8th edition

### Primary outcomes: 90-day mortality and major morbidity

**Fig. 2:**
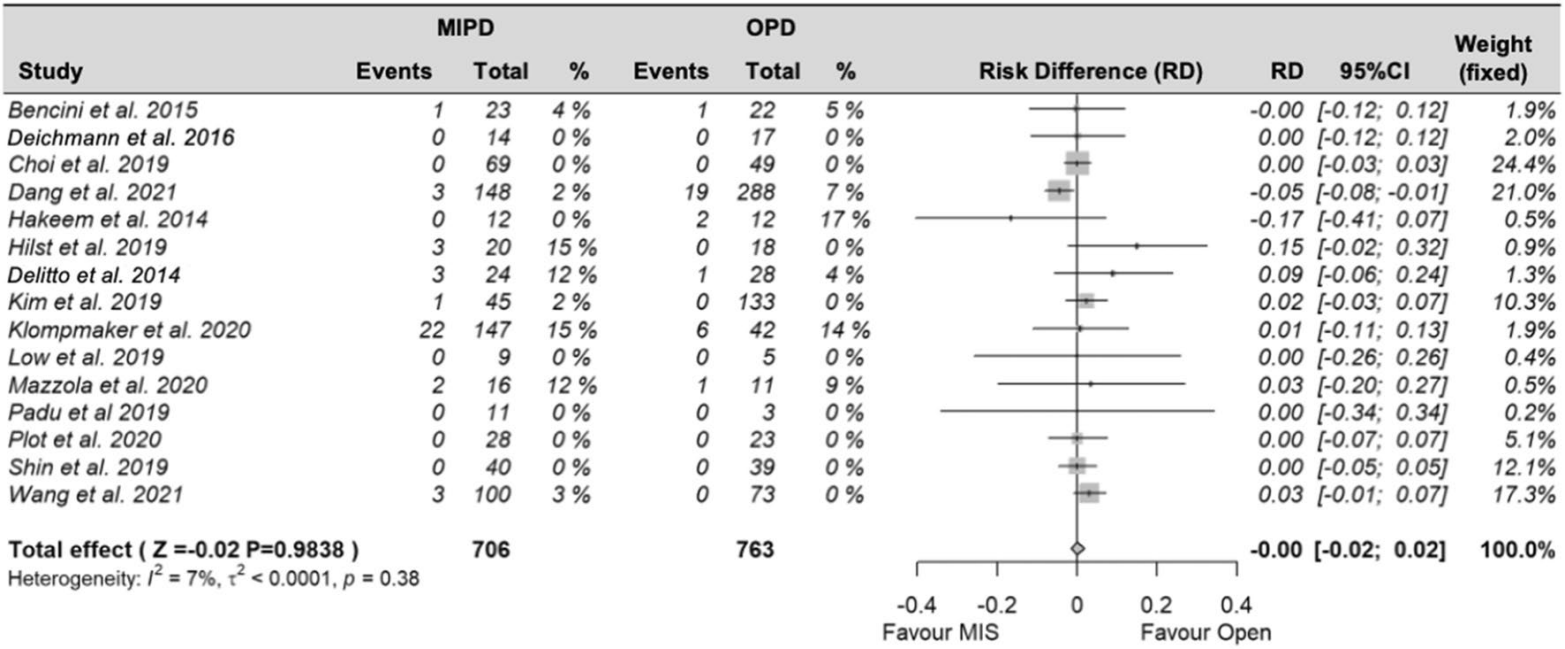
Meta-analysis of 90-day mortality between MIPD and OPD. Abbreviations: MIPD, minimally invasive pancreatoduodenectomy; OPD, open pancreatoduodenectomy; RD, risk difference; 95% CI, confidence interval

**Fig. 3:**
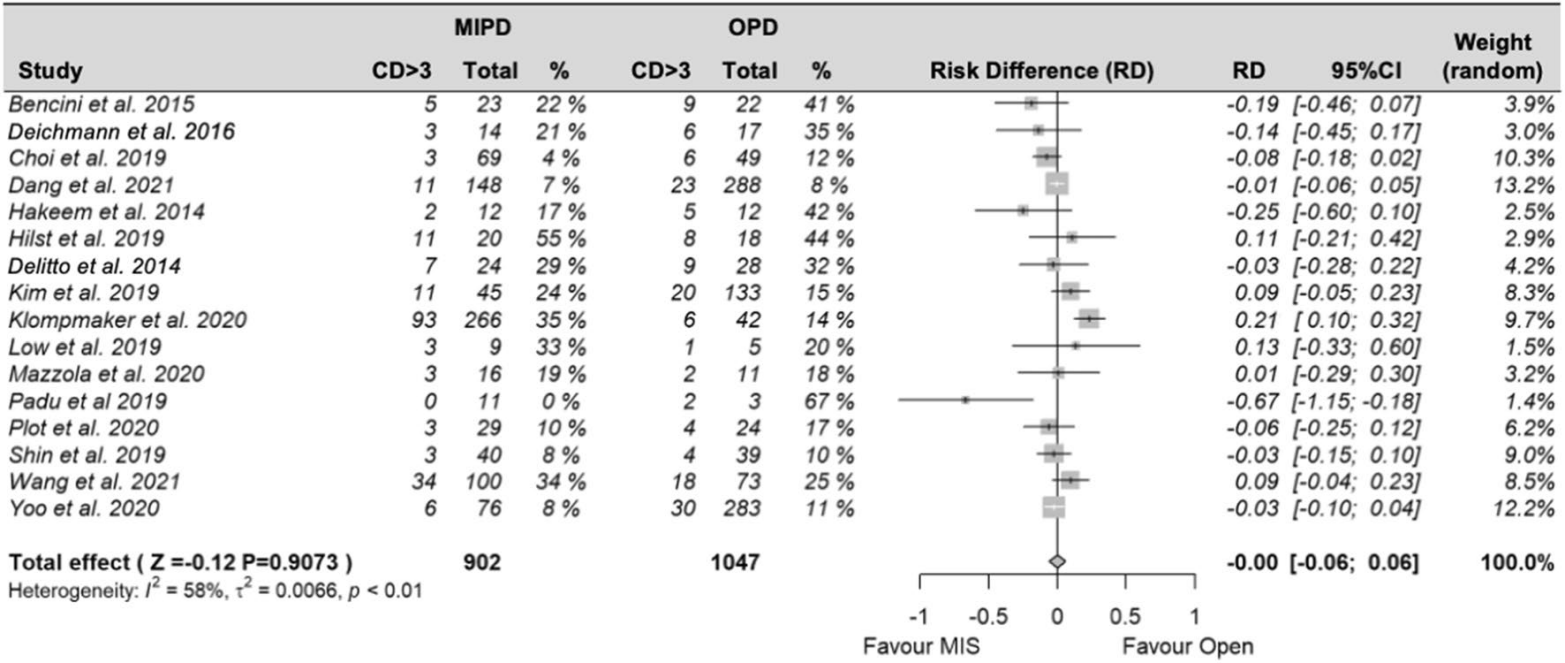
Meta-analysis of major morbidity (Clavien-Dindo 3a – 5) between MIPD and OPD. Abbreviations: CD > 3, Clavien-Dindo 3a-5; MD, mean difference; SD, standard deviation; 95% CI, confidence interval

Overall, 15 studies reported on 90-day mortality, and 16 studies reported on major morbidity. Meta-analysis showed no difference in 90-day mortality (RD -0.01; 95% CI − 1.69–1.66%; *P* = 0.984; Fig. 2) and major morbidity (RD − 0.04%; 95% CI -6.4–5.7; *P* = 0.907; Fig. 3) between MIPD and OPD, respectively. More detailed analyses are shown in the Appendix (Fig. A2-A4).

### Secondary outcomes: perioperative

**Fig. 4:**
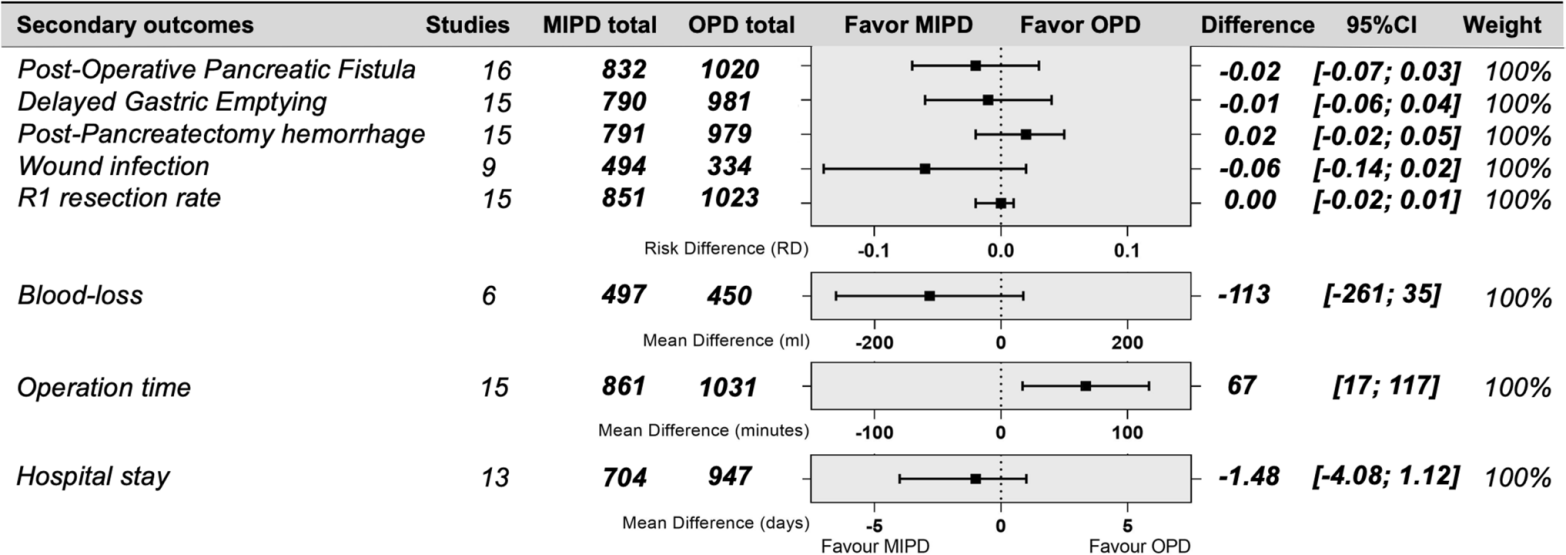
Summary of the meta-analysis with the risk difference or mean difference of the included studies combined. Displayed ranges correlate with the 95% confidence interval. Operation time significance, *P* = 0.009. Abbreviations: MIPD, minimally invasive pancreatoduodenectomy; OPD, open pancreatoduodenectomy; POPF, postoperative pancreatic fistula; DGE, delayed gastric emptying; PPH, postpancreatectomy hemorrhage; WI, wound infections; R1, resection margin < 1 mm

Six studies reported on blood-loss. Median blood-loss for MIPD was 200 ml (IQR 100–300 ml) compared to median of 300 ml (IQR 200–500 ml) for OPD. The MD was − 113 ml for the MIPD cohort (95% CI − 261–35 ml; *P* = 0.135; Fig. 4). In total, 15 studies reported on operative time. The operation time for MIPD was on average 383 min versus 336 min for OPD with a MD of + 67 min (95% CI 17–117 m; *P* = 0.009; Fig. 4) in the MIPD group. More detailed analyses are shown in the Appendix (Fig. A5-A6).

### Secondary outcomes: postoperative

All studies reported on postoperative complications [29, 33, 51, 52, 58–64, 66–69]. The incidence rate of the following complications did not differ between MIPD and OPD: POPF (RD − 1.7; 95% CI − 6.8–3.5%; *P* = 0.524; Fig. 4), DGE (RD − 1.0; 95% CI − 5.6–3.6%; *P* = 0.678; Fig. 4), PPH (RD 1.7%; 95% CI − 2.0–5.3%; *P* = 0.369; Fig. 4), and wound infections (RD − 6.0%; 95% CI − 13.6–1.6%; *P* = 0.120; Fig. 4). MIPD was not associated with a significantly shorter hospital stay (MD − 1.5 days; 95% CI − 4.1–1.1 days, *P* = 0.266; Fig. 4). Fifteen studies reported the R0/R1 resection rate [29, 33, 51, 52, 58–64, 66–69]. The R1 resection rate did not differ significantly between MIPD and OPD with a RD of − 0.4% for MIPD (95% CI − 2.1–1.3%; *P* = 0.624; Fig. 4). More detailed analyses are shown in the Appendix (Appendix, Fig. A7-A12).

### Subgroup analyses

**Fig. 5:**
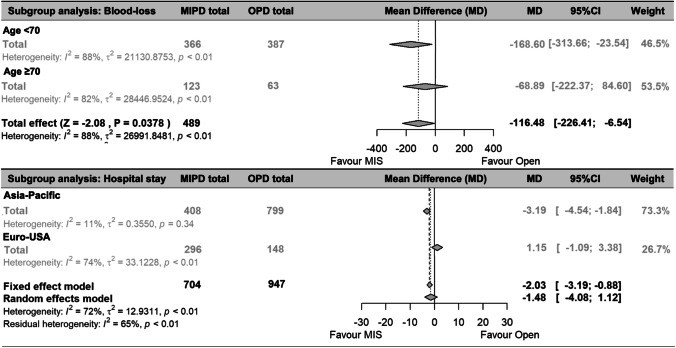
Subgroup analysis Blood-loss (in cc) and Hospital stay (in days). Abbreviations: MIPD, minimally invasive pancreatoduodenectomy; OPD, open pancreatoduodenectomy; MIS, minimally invasive surgery; MD, mean difference; 95% CI, confidence interval

The main subgroup analysis was to compare MIPD and OPD for the three NPPCs separately. The 90-day mortality and major morbidity were comparable between MIPD and OPD for ampullary, distal cholangio, and duodenal cancer. Due to the differences in perioperative blood-loss and the use of oral anticoagulant drugs, subgroup analysis for age was performed and showed that MIPD is associated with significantly less blood-loss for the patients below 70 years old (MD − 169 ml; 95% CI − 314 to − 24 ml; *P* = 0.023; Fig. 5). For the patients with an age above 70, the measured reduction in blood-loss (− 69 ml) was not significant (95% CI − 222–84 ml, Fig. 5). Also, due to differences in postoperative care, geographic location of the center is analyzed for its influence on the length of hospital stay. In Eastern centers, MIPD was associated with a significant reduction in hospital stay of 3.2 days (95% CI − 4.7 to − 1.7 days, *P* < 0.001; Fig. 5) compared to OPD. Furthermore, subgroup analyses between pylorus-preserving and pylorus-resecting pancreatoduodenectomy, between Western and Eastern centers, and between young and elderly patients did not result in significant differences in 90-day mortality and major morbidity (Appendix, Fig A3 and A4). In addition, there are no significant differences found in 90-day mortality, major mortality, or POPF when compared OPD with laparoscopic PD or robotic PD separately (appendix, Fig A14).

### Disease-free and overall survival

**Fig. 6:**
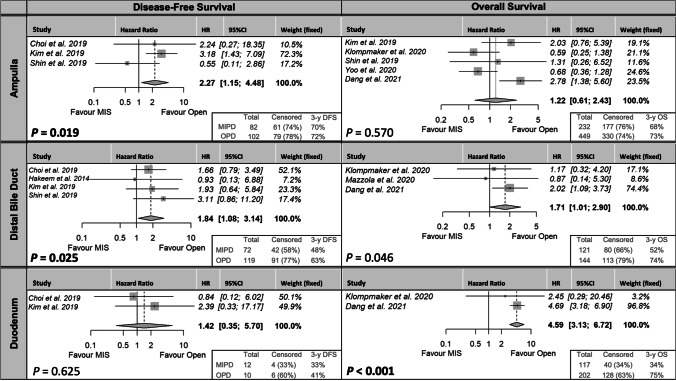
Disease-free survival (left) and overall survival (right). Abbreviations: MIPD, minimally invasive pancreatoduodenectomy; OPD, open pancreatoduodenectomy; HR, hazard ratio for MIPD compared to OPD; 95% CI, confidence interval; 3-y DFS, 3-year disease-free survival in percentage

Four studies reported on DFS [33, 58, 63, 67], and five studies reported on OS [29, 33, 51, 63, 64]. DFS was longer in the OPD group for ampullary (3-year DFS 70 versus 72%; HR 2.27; 95% CI 1.15–4.48; *P* = 0.019; Fig. 6) and distal cholangio (3-year DFS 48 versus 63%; HR 1.84; 95% CI 1.08–3.14; *P* = 0.025; Fig. 6), but not for duodenal cancer (3-year DFS 33 versus 41%; HR 1.42; 95% CI 0.35–5.70; *P* = 0.625; Fig. 6). The number of patients included for analyses was limited, and there were less patients in the MIPD group for ampullary (82 versus 102), distal cholangio (72 versus 119), but not for duodenal (10 versus 12) cancer. Also, there were less patients censored in the MIPD group for ampullary (74 versus 78%), distal cholangio (58 versus 77%), and duodenal (33 versus 60%). The OS was longer in the OPD group for distal cholangio (3-year OS 52 versus 74%; HR 1.17; 95% CI 1.01–2.90; *P* = 0.046; Fig. 6) and duodenal cancer (3-year OS 34 versus 75%; HR 4.59; 95% CI 3.13–6.72; *P* =  < 0.001; Fig. 6), but not for ampullary cancer (3-year OS 68 versus 73%; HR 1.22; 95% CI 0.61–2.43; *P* = 0.570; Fig. 6). For the OS analyses as well, there were less patients in the MIPD group for ampullary (232 versus 449), distal cholangio (121 versus 144), and for duodenal (117 versus 202) cancer. Also, there were less patients censored in the MIPD group for distal cholangio (66% versus 79%) and duodenal (34 versus 63%) cancer. Only for ampullary cancer, the number of censored patients was comparable (76 versus 74%).

### Publication bias

Funnel plots were created for each outcome and assessed for symmetry. There was no asymmetry found in the funnel plots for the primary outcomes of 90-day mortality and major morbidity, suggesting no or limited publication bias (Appendix Fig. A15). Also, the funnel plots for POPF, DGE, PPH, hospital stay, blood-loss, operation time, or R1 resections were symmetrical. However, only the funnel plot for the wound infections is significantly different from zero (Egger’s test: *P* = 0.018).

## Discussion

This first IPDMA on minimally invasive versus open pancreatoduodenectomy for NPPC (ampullary, distal cholangio, and duodenal cancer) found that MIPD is associated with comparable 90-day mortality, major morbidity, and postoperative complications compared to OPD. Subgroup analyses show that MIPD was associated with a shorter hospital stay in Eastern centers, and MIPD was associated with less blood-loss in patients below the age of 70. Moreover, operating time was 67 min longer in the MIPD group. Since there is a lack of data on the impact of MIPD for NPPC in terms of long-term survival, conclusions derived from the existing literature are inaccurate.

Various studies suggested comparable short-term short-term mortality and major morbidity between MIPD and OPD [70]. This study as well shows comparable 90-day mortality and major morbidity after MIPD compared to OPD for NPPC collectively but also for ampullary, distal cholangio, and duodenal cancer separately. Furthermore, minimally invasive surgery is considered to be associated with less trauma and less physical stress for the patient. Therefore, it is believed that the application of MIPD has a potential to reduce major complications. Some studies reported a shorter hospital stay, fewer wound infections, and less blood-loss after MIPD [31, 62, 71, 72]. In this study, postoperative complications are found to be comparable between MIPD and OPD for all NPPCs collectively and for ampullary, distal cholangio, and duodenal cancer separately.

In the current literature, subgroup analyses in the comparison of MIPD and OPD are marginally addressed. The NPPC subgroup was not predicted to influence short-term complications, which this meta-analysis did not contradict by demonstrating similar short-term outcomes for ampullary, distal cholangio, and duodenal cancer. Adequate patient selection, such as defining high-risk groups, is key in improving outcomes in pancreatic surgery. Elderly patients are often classified as high-risk group due to a lack of research selectively focusing on elderly patients [37]. Therefore, some surgeons remain reluctant to adopt the minimally invasive approach in this population [63]. Upon this topic, this study performed subgroup analyses for patients with an age above and below 70 and found similar short-term survival and perioperative outcomes within a large number of patients. Notably, MIPD resulted in significantly lower blood-loss than OPD among patients under 70 years old, while the blood-loss rates were comparable for patients aged 70 years and above. It is known that elderly patients generally have more perioperative blood-loss during general surgery [37] and more frequently use oral anticoagulant drugs. A well-known benefit of minimally invasive surgery is a reduction in blood-loss, which may be less prominent in elderly patients or those on anticoagulant medications. Nonetheless, the results of this study indicate that MIPD can be offered safely to patients over the age of 70 in terms of mortality and postoperative complications, comparable to OPD when performed by an experienced surgeon. Furthermore, subgroup analyses of the geographic location of the performing center were applied in the evaluation of hospital stay. This subgroup analysis was required considering that hospital stay can be longer in Eastern centers due to the goal of discharging the patients to their previous living situation instead of the option for a temporary rehabilitation clinic or intensive medical home care. Indeed, our cohort showed a longer hospital stay in Eastern centers as well for MIPD and OPD. In addition, some studies suggested a decreased length of hospital stay for MIPD [29, 33] which is supported by this study for patients in Eastern centers. The difference in length of hospital stay between MIPD and OPD in Western centers was not significant. A potential explanation could be that the benefit of MIPD (less surgical trauma and stress, early mobilization) plays an essential factor in a later phase of the rehabilitation of the patients when some of the Western patients already have been discharged to a rehabilitation clinic or medical home care. Therefore, the benefit of the MIPD on the length of hospital stay will be greater in centers who keep patients admitted until further rehabilitation. This insinuates that the improvements in Western centers should result in shorter stay in rehabilitation centers and should be measured in time to functional recovery or time to return to previous living situation to find the true effect of MIPD on postoperative recovery, which should be confirmed in future studies.

Due to differences in biological behavior of the NPPC subgroups, long-term results are essential. Survival and recurrence rates for MIPD versus OPD for all periampullary tumors are reported to be comparable by multiple studies [32, 33, 57, 66–68, 73] or even improved by the minimally invasive approach for DFS [67, 73] or OS [66]. However, literature on long-term oncological outcomes for patients with resected NPPC is scarce, and when DFS or OS are analyzed, either the number of patients or the follow-up period was limited [29, 33, 57, 61, 64]. In addition, long-term outcomes were only assessed for patient cohorts combining all periampullary tumors or cohorts selectively including patients with PDAC while no studies assessed long-term outcomes for ampullary, distal cholangio, or duodenal cancer separately. Therefore, it is unclear if the outcomes for a patient with NPPC can be extrapolated from the current literature. This is the first study that performed a meta-analysis for all NPPCs separately. Surprisingly, in this study, the MIPD group showed a decrease in DFS for ampullary and distal cholangio cancer, as well as a decrease in OS for distal cholangio and duodenal cancer compared to the OPD group. However, these results are insufficient for permanent conclusions since there are potential explanations for impaired outcomes of MIPD in the databases of the available studies. First, the survival and recurrence data of only 4 studies were available in which both the follow-up period and number of patients included were limited in these studies [58]. One study reported exclusively on 30- and 90-day survival [33], the OS for duodenal cancer is 97% based on one study [29], and the DFS of duodenal cancer is only based on 22 patients. Second, more cases were censored in the OPD group that potentially underestimated the number of events (death or recurrence) in this group. Third, there were differences in important predictive variables in the baseline characteristics [12, 74, 75]. The MIPD group has a substantial higher T-stage, BMI, and age. Nonetheless, it was not possible to propensity score the groups or to correct for risk factors associated with survival and recurrence. Nonetheless, the majority of studies comparing MIPD and OPD that reported the inclusion of ampullary, distal cholangio, or duodenal cancers were included in this review. Therefore, these findings based on widely varied, limited in number and follow-up, marginally comparable data of only a few studies indicate an essential research gap for MIPD long-term outcomes in NPPC subgroups.

Some limitations of the current study should be addressed. First, MIPD requires a long learning curve [76], so very early cases may reduce the treatment effect. Therefore, in this meta-analysis, studies before 2015 were excluded to prevent all studies were conducted after 2015 in order to lower potential bias resulting from the learning curve. Yet, it was challenging to assess if the analyzed databases did not include patients in the early phase of the learning curve. Most studies sought to minimize the effect of the learning curve by including only patients operated by one [29, 52, 58, 60, 61, 66, 68] or two [62] experienced MIPD surgeon(s) with a surpassed learning curve. Studies who included patients operated by different surgeons required an annual volume of > 10 MIPDs [64] or a personal experience of the surgeon of > 20 MIPDs [59], > 30 MIPDs [33], or even > 104 MIPDs [69], and two of the included studies did not report how they prevented the results from the bias of the learning curve [63, 77]. Therefore, it is likely to assume that the included OPD patients in this study were in general operated in a further phase of the surgeon learning curve compared to the MIPD cases which resulted in a disadvantage for MIPD in the analyses. Second, the retrospective aspect of most included studies in surgical techniques introduces the risk of selection bias. Different surgeons prefer different techniques based on their experience. The large numbers of included patients in this review and the inclusion of both randomized controlled trials and cohort studies will minimalize this bias. However, it remains practically impossible to exclude selection in the retrospective setting. Third, the data is collected in multiple centers. Therefore, it was difficult to validate the data on their individual quality. Only events and complications reported by the providing center could be included, resulting in a potential underestimation of the exact number of complications. All received databases were validated with other centers. Studies with deviating data and results were asked to review the database again. Fourth, the international multicontinental design resulted in a collection of centers with sociocultural differences in their healthcare systems. It is possible that this results in differences in postoperative treatment protocols and could have affected the results. Fifth, there was a variation found in R1 resection rate among the included studies. This can be due to either the absence of clear definitions in the studies included or the lack of standardized pathology reporting for the resection margins. In order to ensure oncological safety, future studies should use a uniform definition and implement standardized pathological examinations. Sixth, this study comparing MIPD and OPD did not specifically explore the impact of periampullary tumor differences on surgical outcomes; future research should investigate this aspect to improve understanding of the factors influencing variations in outcomes.

Within these limitations, the principal strength of the present study is the IPDMA design, which is the first in assessing the surgical approach for NPPC and its subgroups. Due to this approach, this is the first study that could assess the different NPPC subgroups. Also, regardless of the rarity of the NPPC tumors, this IPDMA reached large numbers of patients. This allowed to evaluate subgroups and efficiently assess more subtle differences in subgroups between the minimally invasive and open approach for the selected patients. Moreover, 16 centers delivered their database (some of which are responsible for multiple studies in the field), resulting in a database including practically all of the important studies on the topic and thus a valid representation of all published cases and available evidence to date.

In conclusion, this systematic review and IPDMA suggest a safe implication of MIPD only in the perioperative and postoperative period for patients with NPPC. However, the available long-term data suffer from several major limitations which highlight an essential research gap that should be investigated in prospectively maintained international registries or cohort studies with longer follow-up periods for ampullary, distal cholangio, and duodenum cancer separately.

### Supplementary Information

Below is the link to the electronic supplementary material.

Supplementary file1 (DOCX 56434 KB)

## Data Availability

Data used for this study is not available online. For requests, the corresponding author should be contacted.

## Abbreviations

AJCC: American Joint Committee on Cancer
ASA: American Society of Anesthesiologists
DFS: Disease-free survival
DGE: Delayed gastric emptying
HR: Hazard ratio
IPDMA: Individual Patient Data Meta-Analysis
IQR: Inter quartile range
MD: Mead difference
MIPD: Minimally invasive pancreatoduodenectomy
NPPC: Non-pancreatic periampullary cancer
OPD: Open pancreatoduodenectomy
OS: Overall survival
PD: Pancreatoduodenectomy
PDAC: Pancreatic ductal adenocarcinoma
POPF: Postoperative pancreatic fistula
PPH: Postpancreatectomy hemorrhage
RD: Risk difference
SD: Standard deviation
SMD: Standardized mean difference
