# Supplementary material for: The clinical implication of minimally invasive versus open pancreatoduodenectomy for non-pancreatic periampullary cancer: a systematic review and individual patient data meta-analysis
Source: Langenbecks Arch Surg. 2023 Aug 15;408(1):311. doi: 10.1007/s00423-023-03047-4 (PMC10427526; doi:10.1007/s00423-023-03047-4)
Supplement: Supplementary file 1 — Supplementary file1 (DOCX 56434 KB) [file 423_2023_3047_MOESM1_ESM.docx]

**Appendix**

**
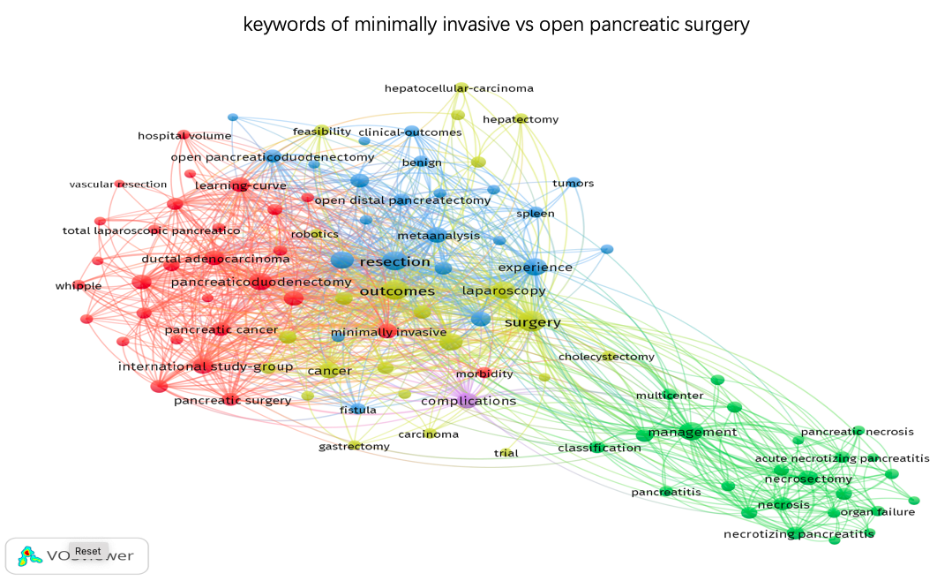
**

**Figure A1:** Bibliometric analysis of minimally invasive vs open pancreatic surgery from Web of Science in 890 articles until 31/12/2021. As shown, widely investigated topics are the surgical techniques and complications. For the different etiology only the pancreatic ductal adenocarcinoma is found, hence a lack in publications regarding different non-pancreatic peri-ampullary subtypes.

**Full web search:**

Medline (via Ovid):

Database(s): Ovid MEDLINE(R) and Epub Ahead of Print, In-Process, In-Data-Review & Other Non-Indexed Citations and Daily 1946 to December 31, 2021
Search Strategy:

| **#** | **Searches** | **Results** |
| --- | --- | --- |
| 1 | exp Robotics/ or exp Surgery, Computer-Assisted/ or exp Robotic Surgical Procedures/ | 49534 |
| 2 | (robot or robotic surgery).mp. | 27047 |
| 3 | robotics.ti,ab. | 6814 |
| 4 | robotic surgery.ti,ab. | 5450 |
| 5 | exp Laparoscopy/ | 104054 |
| 6 | laparoscopic.ti,ab. | 114739 |
| 7 | laparoscopy.ti,ab. | 36040 |
| 8 | laparoscopic-assisted.ti,ab. | 2702 |
| 9 | open.ti,ab. | 533371 |
| 10 | robotic.ti,ab. | 31936 |
| 11 | robotic-assisted.ti,ab. | 5476 |
| 12 | laparoscopy-assisted.ti,ab. | 1636 |
| 13 | robot-assisted.ti,ab. | 9547 |
| 14 | 1 or 2 or 3 or 4 or 5 or 6 or 7 or 8 or 9 or 10 or 11 or 12 or 13 | 706568 |
| 15 | exp Pancreaticoduodenectomy/ | 8639 |
| 16 | Pancreaticoduodenectomy.ti,ab. | 7086 |
| 17 | Whipple surgery.ti,ab. | 54 |
| 18 | Whipple procedure.ti,ab. | 801 |
| 19 | pylorus-preserving pancreaticoduodenectomy.ti,ab. | 544 |
| 20 | duodenopancreatectomy.ti,ab. | 657 |
| 21 | pancreatectomy.ti,ab. | 9919 |
| 22 | distal pancreatectomy.ti,ab. | 3893 |
| 23 | spleen-preserving pancreatectomy.ti,ab. | 13 |
| 24 | 15 or 16 or 17 or 18 or 19 or 20 or 21 or 22 or 23 | 20577 |
| 25 | 14 and 24 | 2465 |
| 26 | limit 25 to yr="2015 -Current" | 210 |

Embase (via Ovid):

Database(s): Embase Classic+Embase 1947 to 2021 December 31
Search Strategy:

| **#** | **Searches** | **Results** |
| --- | --- | --- |
| 1 | exp robotics/ | 42291 |
| 2 | exp computer assisted surgery/ | 24051 |
| 3 | exp robot assisted surgery/ | 14109 |
| 4 | (robot or robotic surgery).mp. | 47396 |
| 5 | robotics.ti,ab. | 8300 |
| 6 | robotic surgery.ti,ab. | 9335 |
| 7 | exp laparoscopy/ | 172980 |
| 8 | laparoscopic.ti,ab. | 188161 |
| 9 | laparoscopy.ti,ab. | 55693 |
| 10 | laparoscopic-assisted.ti,ab. | 4329 |
| 11 | open.ti,ab. | 715250 |
| 12 | robotic.ti,ab. | 53634 |
| 13 | robotic-assisted.ti,ab. | 11929 |
| 14 | laparoscopy-assisted.ti,ab. | 2237 |
| 15 | robot-assisted.ti,ab. | 16112 |
| 16 | 1 or 2 or 3 or 4 or 5 or 6 or 7 or 8 or 9 or 10 or 11 or 12 or 13 or 14 or 15 | 977825 |
| 17 | exp pancreaticoduodenectomy/ | 23817 |
| 18 | Pancreaticoduodenectomy.ti,ab. | 11517 |
| 19 | Whipple surgery.ti,ab. | 146 |
| 20 | Whipple procedure.ti,ab. | 1470 |
| 21 | pylorus-preserving pancreaticoduodenectomy.ti,ab. | 803 |
| 22 | duodenopancreatectomy.ti,ab. | 932 |
| 23 | pancreatectomy.ti,ab. | 16766 |
| 24 | distal pancreatectomy.ti,ab. | 6786 |
| 25 | spleen-preserving pancreatectomy.ti,ab. | 26 |
| 26 | 17 or 18 or 19 or 20 or 21 or 22 or 23 or 24 or 25 | 38678 |
| 27 | 16 and 26 | 5231 |
| 28 | limit 27 to yr="2015 -Current" | 236 |

[Cochrane Central Register of Controlled Trials](https://eur04.safelinks.protection.outlook.com/?url=https%3A%2F%2Fwww.cochranelibrary.com%2F&data=04%7C01%7Cb.uijterwijk%40amsterdamumc.nl%7Ce6d5ed2cba6f4b67e8a508d9d7715ebb%7C68dfab1a11bb4cc6beb528d756984fb6%7C0%7C0%7C637777704182157238%7CUnknown%7CTWFpbGZsb3d8eyJWIjoiMC4wLjAwMDAiLCJQIjoiV2luMzIiLCJBTiI6Ik1haWwiLCJXVCI6Mn0%3D%7C3000&sdata=MdwFYijN0jtXzZZq7kMLfN7fH3xg6K9LFfoZzKuye8s%3D&reserved=0)

Issue 7 of 12, December 2021

31 results

[Cochrane Database of Systematic Reviews](https://eur04.safelinks.protection.outlook.com/?url=https%3A%2F%2Fwww.cochranelibrary.com%2F&data=04%7C01%7Cb.uijterwijk%40amsterdamumc.nl%7Ce6d5ed2cba6f4b67e8a508d9d7715ebb%7C68dfab1a11bb4cc6beb528d756984fb6%7C0%7C0%7C637777704182157238%7CUnknown%7CTWFpbGZsb3d8eyJWIjoiMC4wLjAwMDAiLCJQIjoiV2luMzIiLCJBTiI6Ik1haWwiLCJXVCI6Mn0%3D%7C3000&sdata=MdwFYijN0jtXzZZq7kMLfN7fH3xg6K9LFfoZzKuye8s%3D&reserved=0)

Issue 7 of 12, December 2021

O results

ID         Search Hits

#1        (robot or robotic surgery or robotics or laparoscopy or laparoscopic or laparoscopic-assisted or open or robotic or robotic-assisted or laparoscopy-assisted or robot-assisted):ti,ab,kw         131644

#2        (pancreaticoduodenectomy or whipple surgery or whipple procedure or pylorus-preserving pancreaticoduodenectomy or duodenopancreatectomy or pancreatectomy or distal pancreatectomy or spleen-preserving pancreatectomy):ti,ab,kw 1687

#3        #1 and #2 with Cochrane Library publication date Between Jan 2015 and Dec 2021          31

After deduplicating: 3.561 studies.

**Identification of studies via databases and registers**

Records removed *before screening*:

Duplicate records removed (n = 3.276)

Records marked as ineligible by automation tools (n = 0)

Records removed for other reasons (n = 0)

Records identified from

Medline (n = 3.110)

Embase (n = 3.696)

Cochrane (n = 30)

n = 6.836

**Identification**

*Studies excluded: (n = 3.479)*

Not in English

Non-comparative (e.g. systematic review, case reports, only laparoscopic)

Studies (title and abstract) screened

n = 3.561

*Studies excluded: (n = 66)*

No NPPC* included

Technical procedures described

Mice / cadaver studies

Selected patients (e.g. only patents with major vascular resection or elderly).

Sought for retrieval and full article assessed for eligibility

n = 82

**Screening**

Studies eligible for analyses and data requested

n = 22

*Studies excluded: (n = 4)*

Did not want to cooperate *(n = 2)*

Did not respond after multiple reminders *(n = 2)*

Studies used same database** (*n = 2)*

Study’s databases received and included in review:

n = 16

**Included**

**Figure A2:** Flowchart systematic websearch; *NPPC, non-pancreatic periampullary cancer; **: When multiple studies used the same database, the complete database of the most recent study was requested.


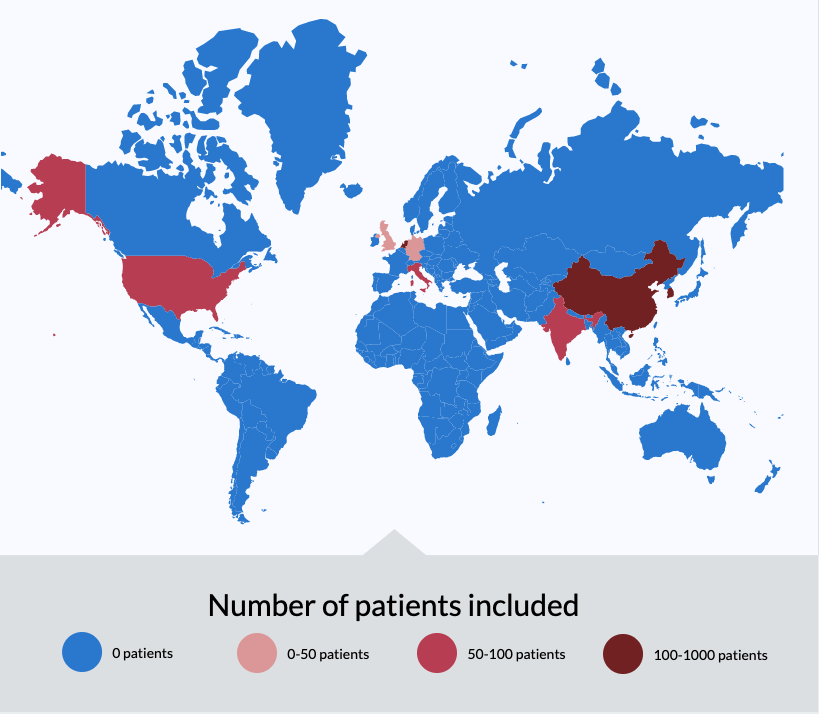


**Figure A3:** World map with geographic origin and number of the included patients.

|  | **Total** | **OPD** | **MIPD** | ***P*-value** | ***SMD*** |
| --- | --- | --- | --- | --- | --- |
| T stage, mean (SD) | *2.3 (0.9)* | *2.2 (0.9)* | *2.4 (0.9)* | **0.001** | 0.195 |
| *AAC, mean (SD)* |  | *2.17 (0.85)* | *2.27 (0.96)* | *0.154* | 0.112 |
| *DCC, mean (SD)* |  | *2.28 (0.67)* | *2.54 (0.70)* | ***0.002*** | **0.357** |
| *DAC, mean (SD)* |  | *2.88 (1.13)* | *3.08 (0.70)* | *0.457* | 0.100 |
|  |  |  |  |  |  |
|  |  |  |  |  |  |
| T stage total, n (%) |  |  |  | **0.006** | 0.174 |
| T1, n (%) |  | *137 (22)* | *92 (18)* |  |  |
| T2, n (%) |  | *239 (38)* | *167 (32)* |  |  |
| T3, n (%) |  | *216 (34)* | *210 (41)* |  |  |
| T4, n (%) |  | *36 (6)* | *47 (9)* |  |  |
|  |  |  |  |  |  |
| *T stage Ampullary*, n (%) |  |  |  | **0.021** | 0.168 |
| T1, n (%) |  | *107 (24)* | *74 (24)* |  |  |
| T2, n (%) |  | *181 (40)* | *115 (37)* |  |  |
| T3, n (%) |  | *137 (31)* | *89 (28)* |  |  |
| T4, n (%) |  | *24 (5)* | *36 (11)* |  |  |
|  |  |  |  |  |  |
| *T stage Distal Cholangio*, n (%) |  |  |  | **0.018** | **0.277** |
| T1, n (%) |  | *26 (19)* | *16 (10)* |  |  |
| T2, n (%) |  | *46 (34)* | *45 (28)* |  |  |
| T3, n (%) |  | *64 (47)* | *97 (60)* |  |  |
| T4, n (%) |  | *0 (0)* | *3 (1)* |  |  |
|  |  |  |  |  |  |
| *T stage Duodenal*, n (%) |  |  |  | **0.035** | **0.625** |
| T1, n (%) |  | *4 (16)* | *1 (4)* |  |  |
| T2, n (%) |  | *5 (20)* | *2 (8)* |  |  |
| T3, n (%) |  | *6 (24)* | *16 (64)* |  |  |
| T4, n (%) |  | *10 (40)* | *6 (24)* |  |  |
|  |  |  |  |  |  |
| Adjuvant Chemotherapy, n (%) | *412 (41)* | *233 (40)* | *179 (43)* | 0.470 | 0.046 |
| *AAC, n (%)* |  | *152 (37)* | *94 (38)* | 0.650 | 0.035 |
| *DCC, n (%)* |  | *72 (50)* | *62 (45)* | 0.394 | 0.102 |
| *DAC, n (%)* |  | *8 (44)* | *23 (61)* | 0.258 | 0.186 |
|  |  |  |  |  |  |
| R1 resection margin,  n (%) | 142 (8) | *75 (7)* | *67 (8)* | 0.659 | 0.020 |
| *AAC, n (%)* |  | *24 (5)* | *14 (4)* | *0.539* | 0.041 |
| *DCC, n (%)* |  | *26 (11)* | *32 (12)* | *0.734* | 0.030 |
| *DAC, n (%)* |  | *24 (9)* | *20 (10)* | *0.852* | 0.018 |
|  |  |  |  |  |  |
|  |  |  |  |  |  |
| PA-positive lymph nodes, median (IQR) |  | 0 (0 – 2) | 0 (0 – 2) | 0.926 |  |
| *AAC, median (IQR)* |  | 0 (0 – 1) | 0 (0 – 1) | 0.503 |  |
| *DCC, median (IQR)* |  | 0 (0 – 2) | 0 (0 – 2) | 0.745 |  |
| *DAC, median (IQR)* |  | 1 (0 – 3) | 0 (0 – 3) | 0.342 |  |
| Lymph node ratio | 0.00  (0.00 – 0.10) | 0.00 (0.00 – 0.10) | 0.00 (0.00 – 0.11) | 0.759 |  |
| *AAC, median (IQR)* |  | 0.00 (0.00 – 0.09) | 0.00 (0.00 – 0.08) | 0.563 |  |
| *DCC, median (IQR)* |  | 0.00 (0.00 – 0.09) | 0.00 (0.00 – 0.16) | 0.362 |  |
| *DAC, median (IQR)* |  | 0.07 (0.00 – 0.19) | 0.00 (0.00 – 0.16) | 0.286 |  |

**Table A1: Extra baseline characteristics of all included patients.**

Abbreviations: SD, Standard deviation; n, count; AC, adenocarcinoma; OPD, open pancreatoduodenectomy; MIPD, minimally invasive pancreatoduodenectomy; SMD, standardized mean difference (not applicable for median and IQR); AAC, ampullary adenocarcinoma; DCC, distal cholangiocarcinoma; DAC, duodenal adenocarcinoma; T stage tumor: differentiated in T1 and T2 vs T3 and T4 groups (elaborate T-stage distribution in supplementary table S3), following AJCC 7^th^ and 8^th^ edition.

| **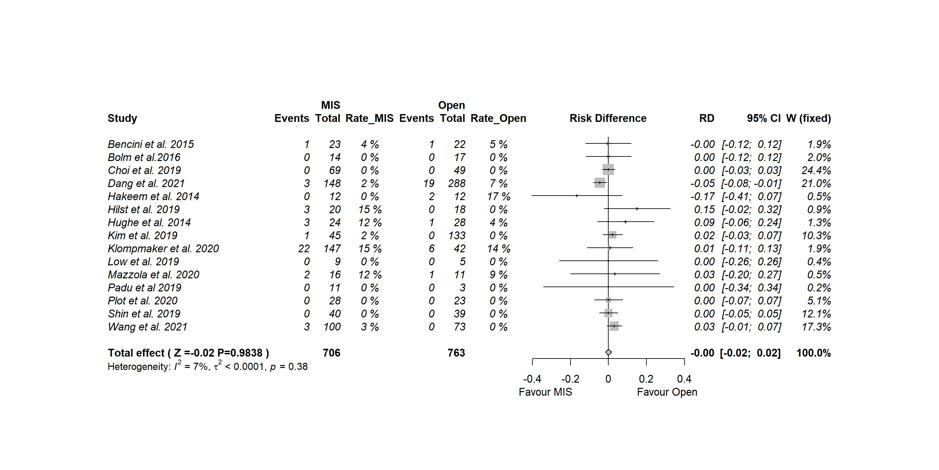** | |
| --- | --- |
| 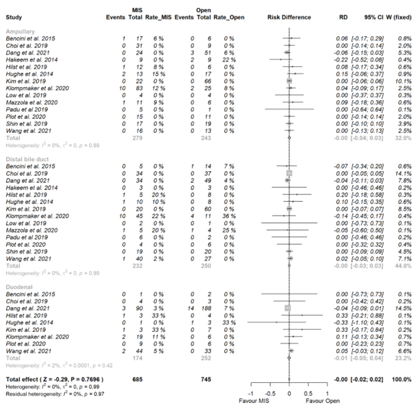 | 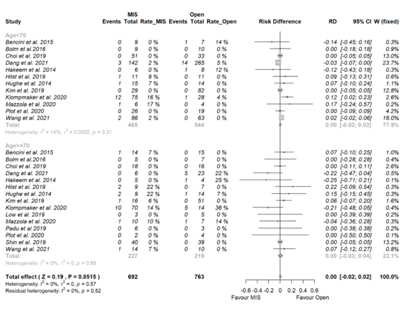 |
| 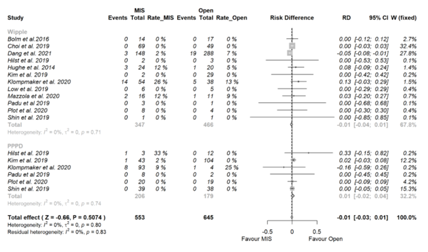 | 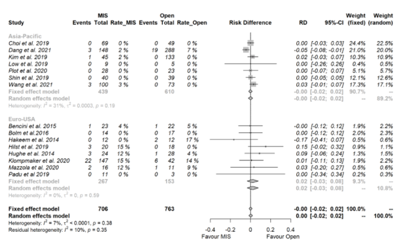 |

**Figure A4:** 90-day mortality above. 90-day mortality subgroup analyses. Middle-above, NPPC subgroups; middle-right, age; left-below, Whipple/pylorus-resecting pancreatoduodenectomy vs pylorus preserving pancreatoduodenectomy (PPPD); right-below, Asia-pacific centers vs European/USA centers.

Abbreviations: RD, Risk Difference; 95%CI, 95% confidence interval.

| **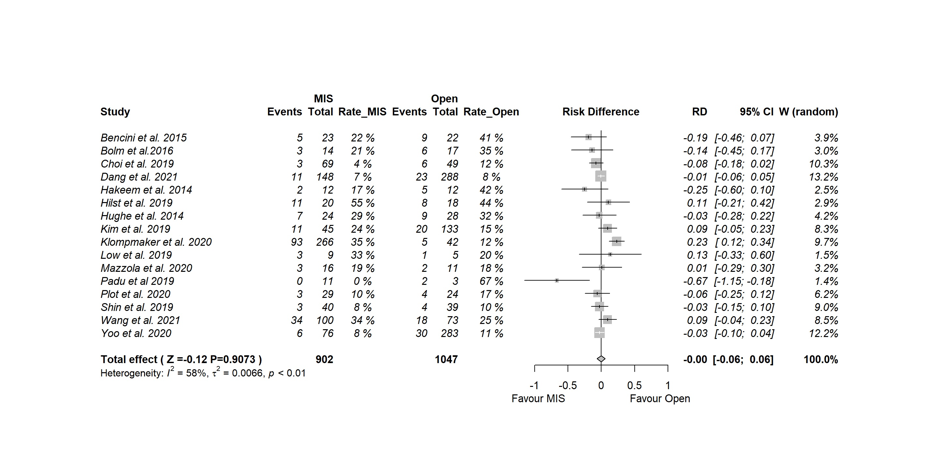** | |
| --- | --- |
| 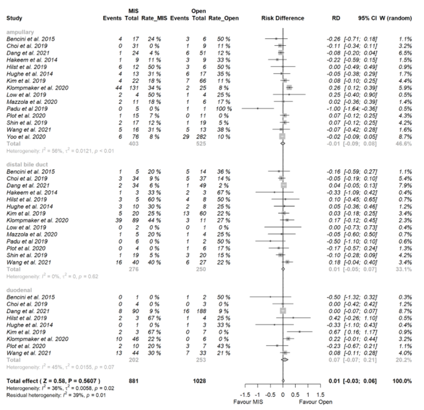 | 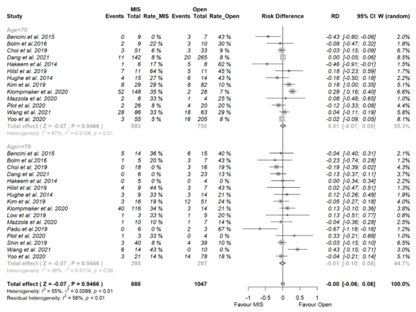 |
| 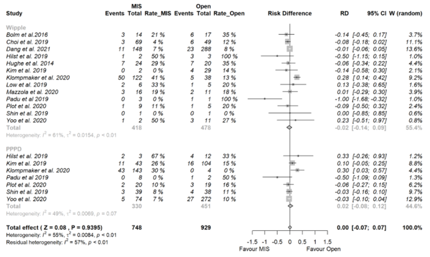 | 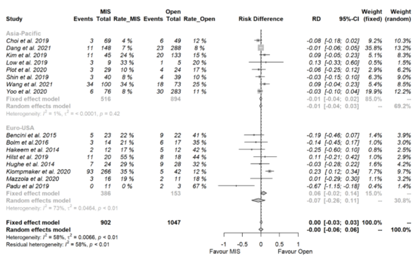 |

**Figure A5:** Major morbidity. Incidence of Clavien Dindo 3a-5 rated complications above. Major Morbidity subgroup analyses. Middle-left, NPPC subgroups; middle-right, age; left-below, Whipple/pylorus-resecting pancreatoduodenectomy vs pylorus preserving pancreatoduodenectomy (PPPD); right-below, Asia-pacific centers vs European/USA centers.

Abbreviations: RD, Risk Difference; 95%CI, 95% confidence interval.

| 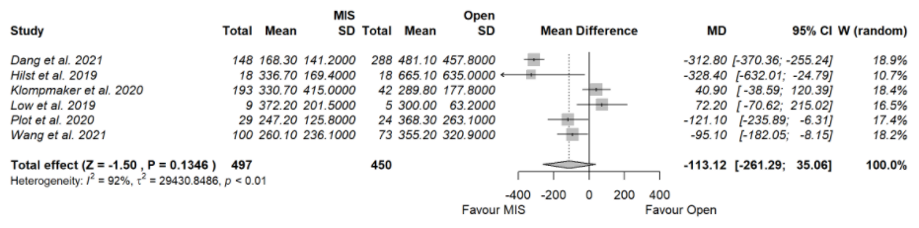 |
| --- |
| 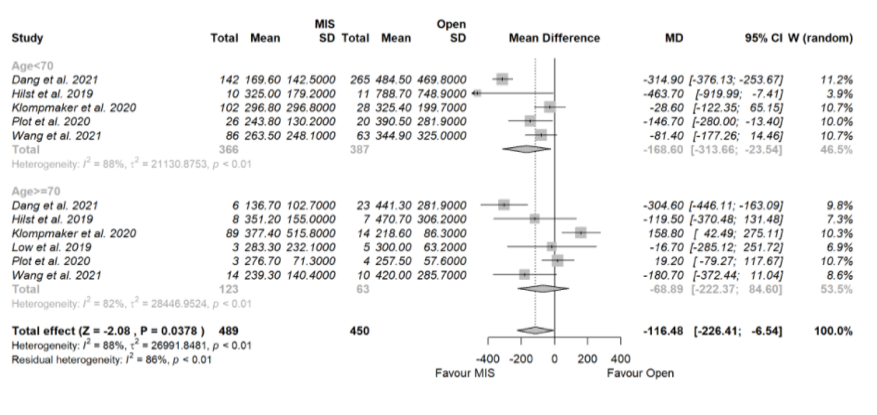 |

**Figure A6:** Per-operative estimated blood-loss (in ml) above. Per-operative estimated blood-loss subgroup analyses for age below.

Abbreviations: MD, mean difference; SD, standard deviation; CI, confidence interval.


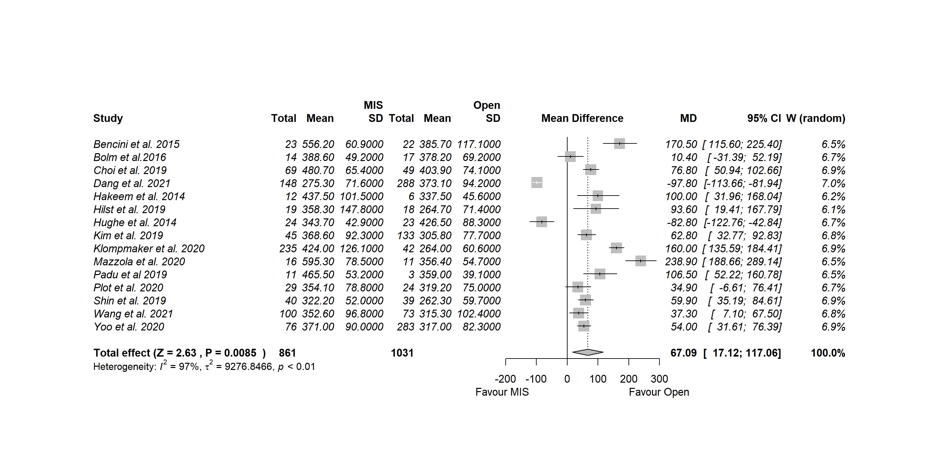


**Figure A7:** Operation time in minutes.

Abbreviations: MD, mean difference; SD, standard deviation; CI, confidence interval.


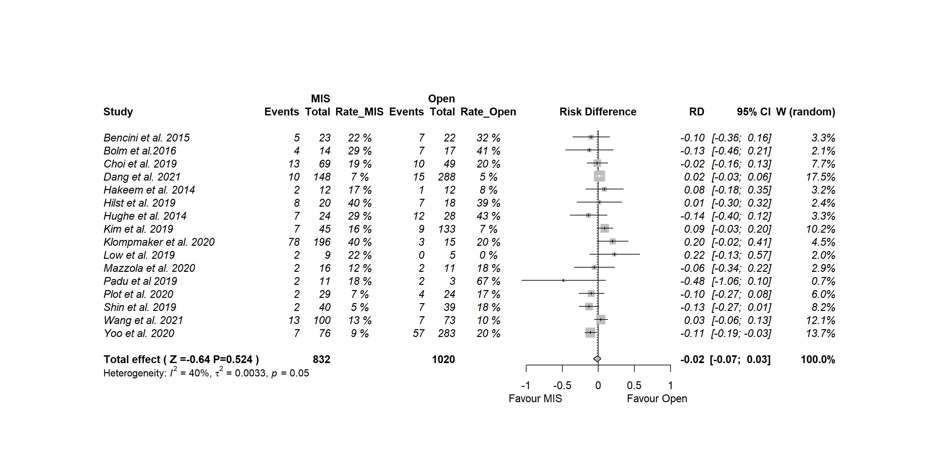


**Figure A8:** Post-operative pancreatic fistula **(**POPF).

Abbreviations: RD, Risk Difference; 95%CI, 95% confidence interval.

| 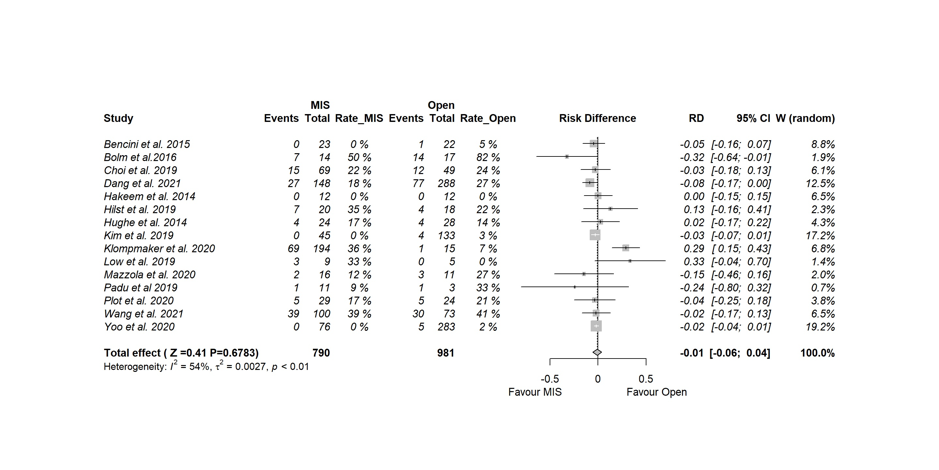 |
| --- |
| 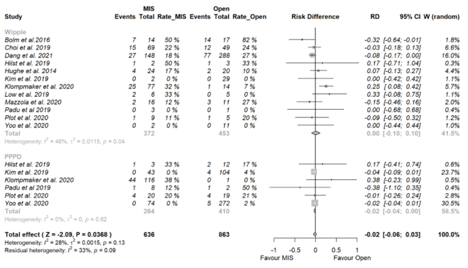 |

**Figure A9:** Delayed Gastric Emptying (DGE) above. Delayed Gastric Emptying (DGE) subgroup analyses below, Whipple/pylorus-resecting pancreatoduodenectomy vs pylorus preserving pancreatoduodenectomy (PPPD)

Abbreviations: RD, Risk Difference; 95%CI, 95% confidence interval.


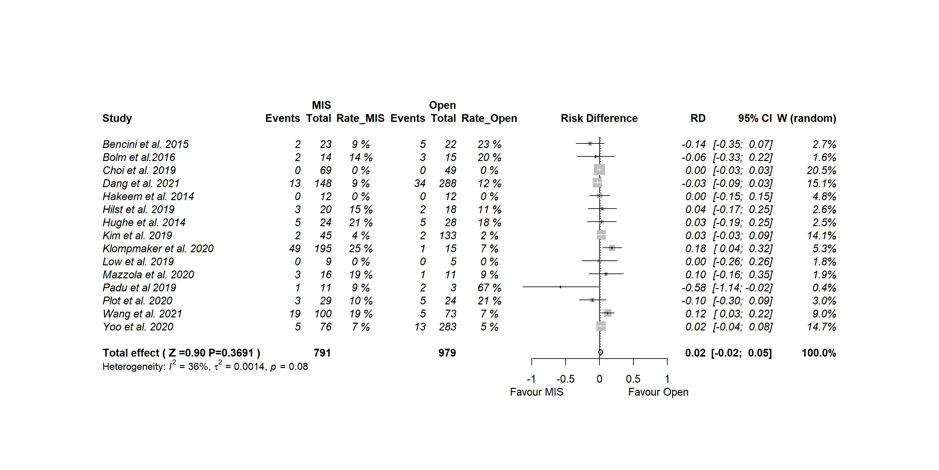


**Figure A10:** Post-pancreatectomy hemorrhage (PPH).

Abbreviations: RD, Risk Difference; CI, confidence interval.


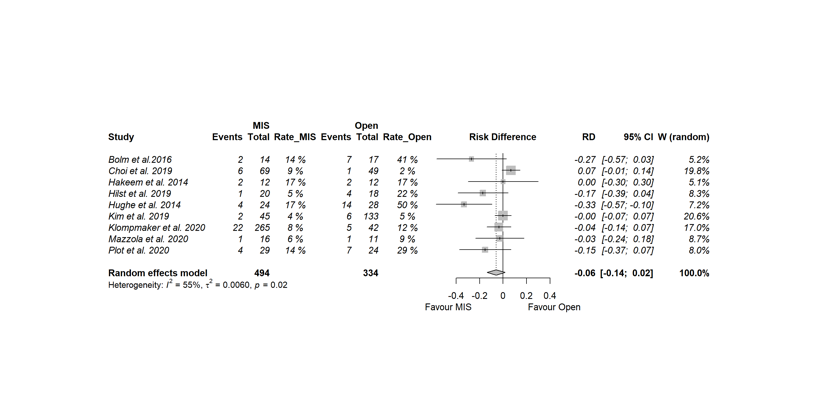


**Figure A11:** Surgical Site Infections (SSI’s).

Abbreviations: RD, Risk Difference; CI, confidence interval.

| 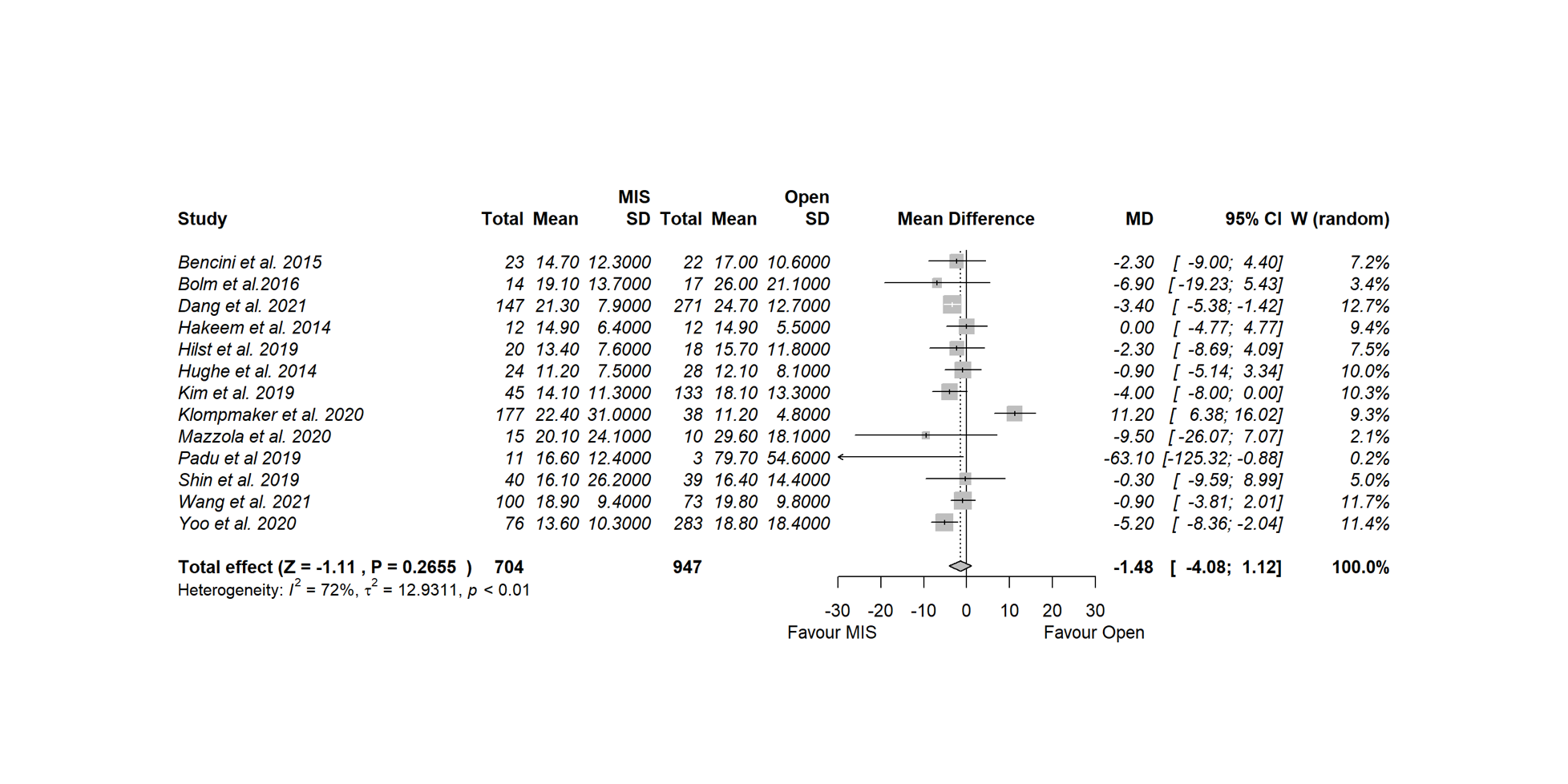 |
| --- |
| 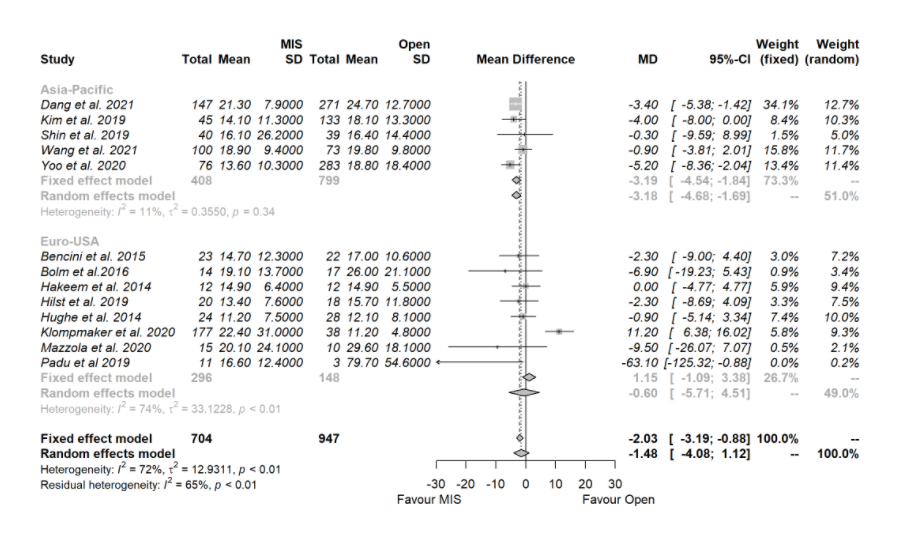 |

**Figure A12:** Length of post-operative hospital stay (LOS) above with subgroup analyses for geographic location of the performing center below.

Abbreviations: MD, mean difference; SD, standard deviation; CI, confidence interval.

**
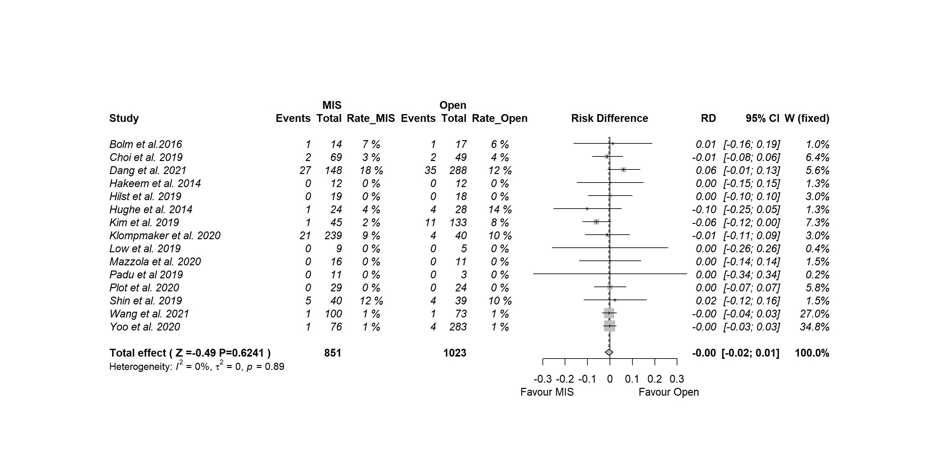
**

**Figure A13:** Incidence of R1 resection margin (<1mm).

Abbreviations: RD, risk difference; CI, confidence interval.

| **A** 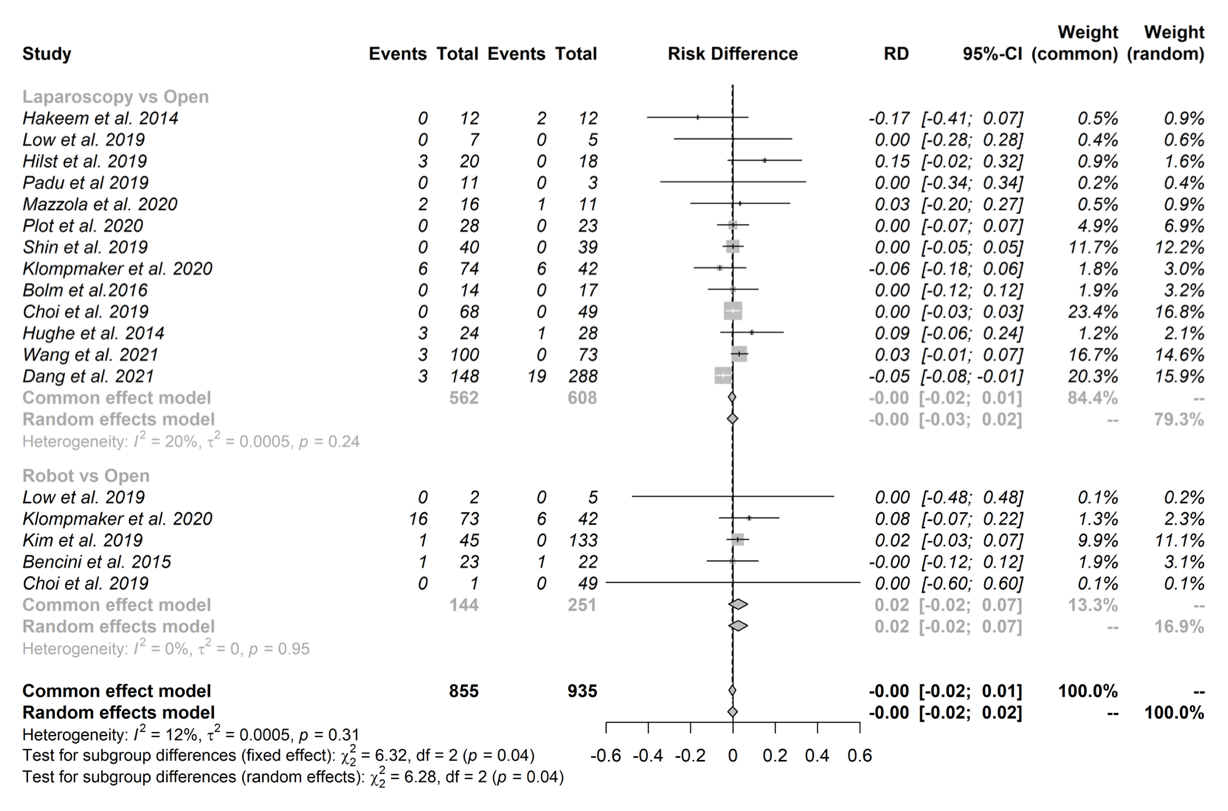 |
| --- |
| **B** **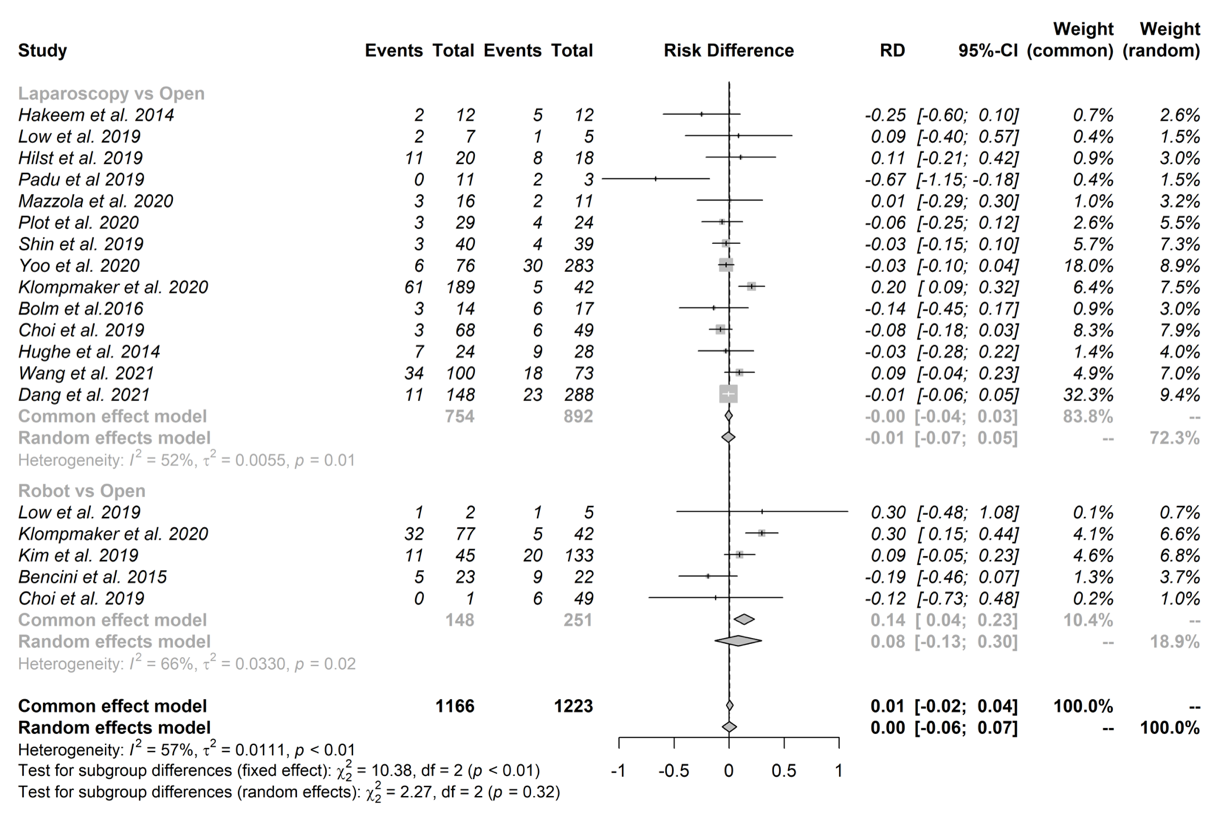** |
| **C** **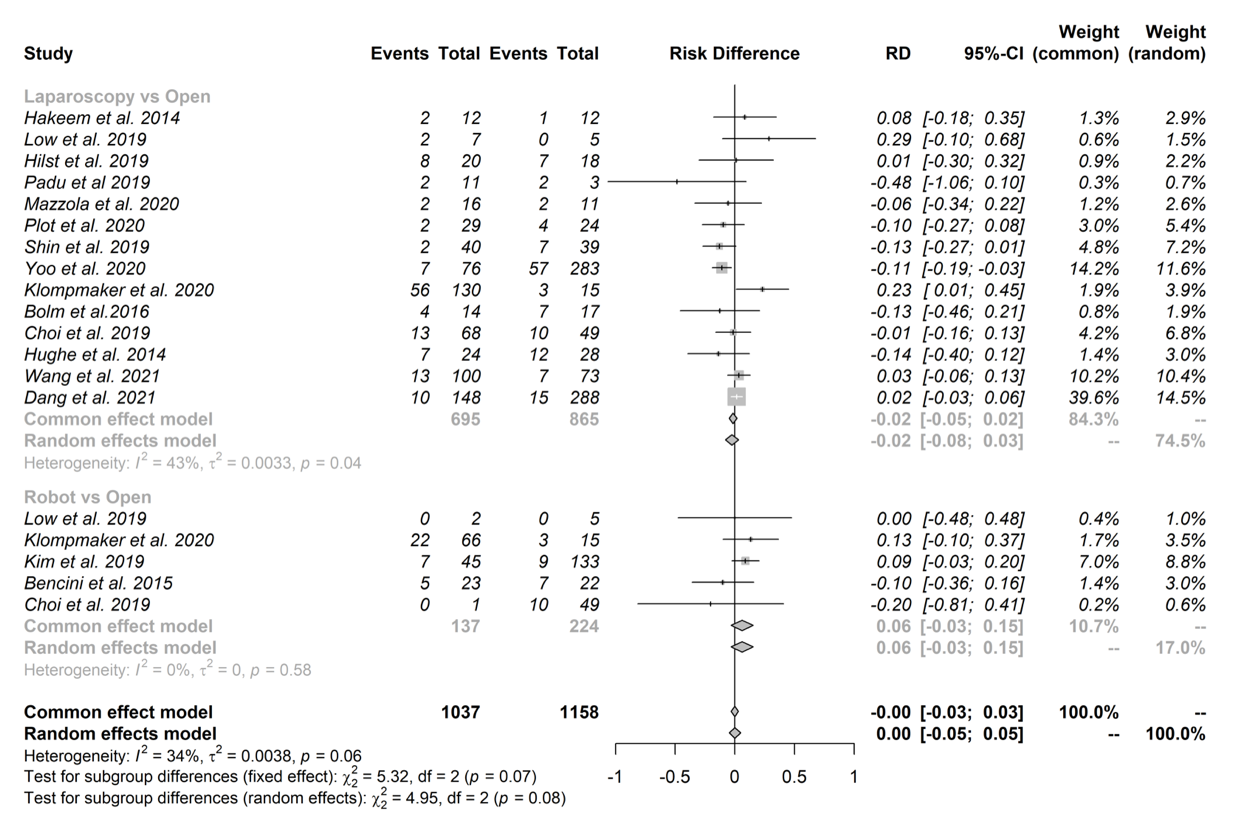** |

**Figure A14:** Subgroup analyses Open PD vs laparoscopic PD and robotic PD for **A** major mortality, **B** major morbidity (Clavien-Dindo 3a-5), and **C** POPF.


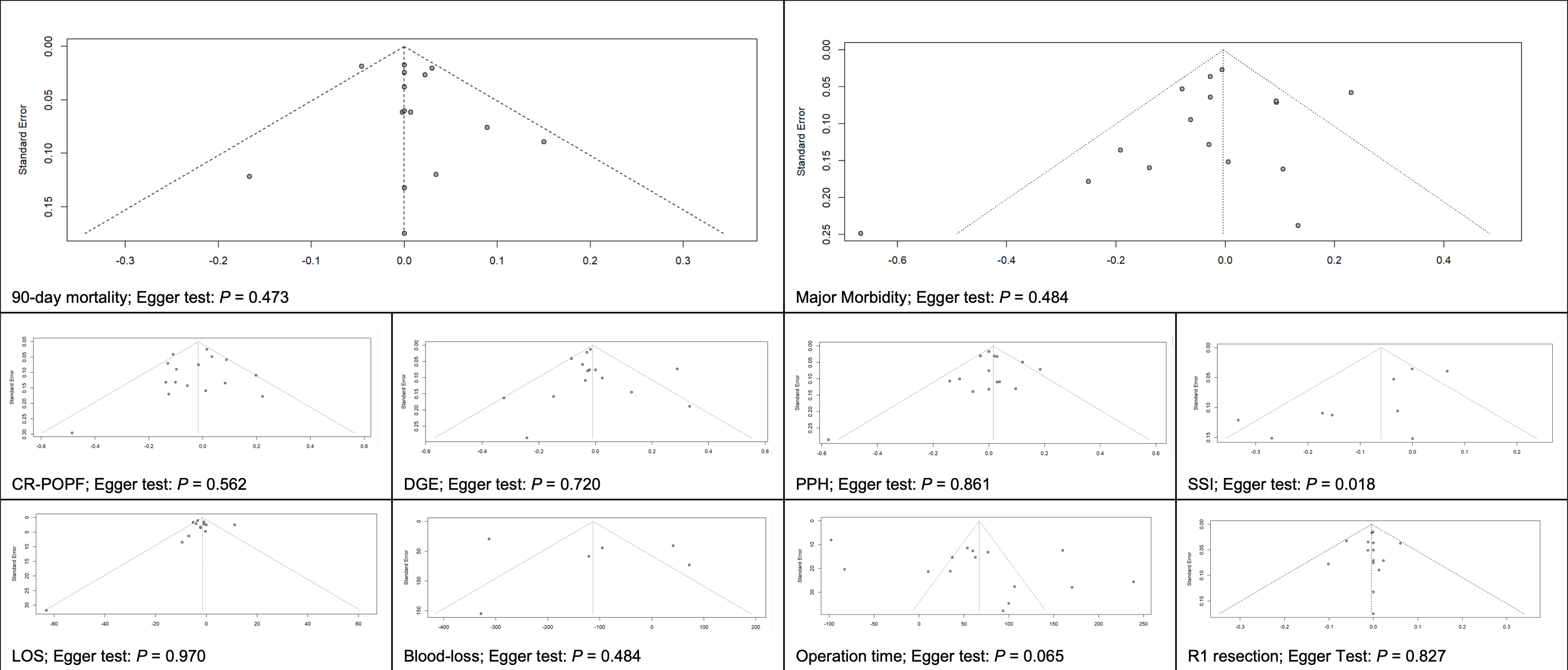


**Figure A15: Funnel plots**
